# Branched-Chain Volatiles in Fruit: A Molecular Perspective

**DOI:** 10.3389/fpls.2021.814138

**Published:** 2022-01-27

**Authors:** Lorenzo N. Bizzio, Denise Tieman, Patricio R. Munoz

**Affiliations:** ^1^Blueberry Breeding and Genomics Lab, Department of Horticultural Sciences, University of Florida, Gainesville, FL, United States; ^2^Plant Molecular and Cellular Biology Program, University of Florida, Gainesville, FL, United States; ^3^Department of Horticultural Sciences, University of Florida, Gainesville, FL, United States

**Keywords:** branched-chain, volatiles, fruit, aroma, flavor, biosynthesis, metabolism

## Abstract

Branched-chain volatiles (BCVs) constitute an important family of fruit volatile metabolites essential to the characteristic flavor and aroma profiles of many edible fruits. Yet in contrast to other groups of volatile organic compounds important to fruit flavor such as terpenoids, phenylpropanoids, and oxylipins, the molecular biology underlying BCV biosynthesis remains poorly understood. This lack of knowledge is a barrier to efforts aimed at obtaining a more comprehensive understanding of fruit flavor and aroma and the biology underlying these complex phenomena. In this review, we discuss the current state of knowledge regarding fruit BCV biosynthesis from the perspective of molecular biology. We survey the diversity of BCV compounds identified in edible fruits as well as explore various hypotheses concerning their biosynthesis. Insights from branched-chain precursor compound metabolism obtained from non-plant organisms and how they may apply to fruit BCV production are also considered, along with potential avenues for future research that might clarify unresolved questions regarding BCV metabolism in fruits.

## Introduction

Volatile organic compounds (VOCs) are essential components of fruit flavor, being necessary for human perception of the distinct flavors produced by the many different types of fruit found in nature (Noble, 1996; Goff and Klee, 2006; El Hadi et al., 2013; Plotto et al., 2017). The importance of VOCs to fruit flavor has prompted researchers to investigate the underlying biosynthetic pathways responsible for their formation. Great progress has been made in understanding the molecular basis of terpenoid (Cheng et al., 2007; Nagegowda, 2010; Tholl, 2015; Abbas et al., 2017), phenylpropanoid (Boatright et al., 2004; Qualley et al., 2012; Lackus et al., 2021), and oxylipin (Mosblech et al., 2009; Scala et al., 2013; Griffiths, 2015; Ameye et al., 2018) volatile biosynthesis in plants. However, much less is known about the molecular correlates underlying the production of branched-chain volatiles (BCVs), a family of compounds encompassing some VOCs that are notable contributors to the flavor of several important fruit crops.

**FIGURE 1:**
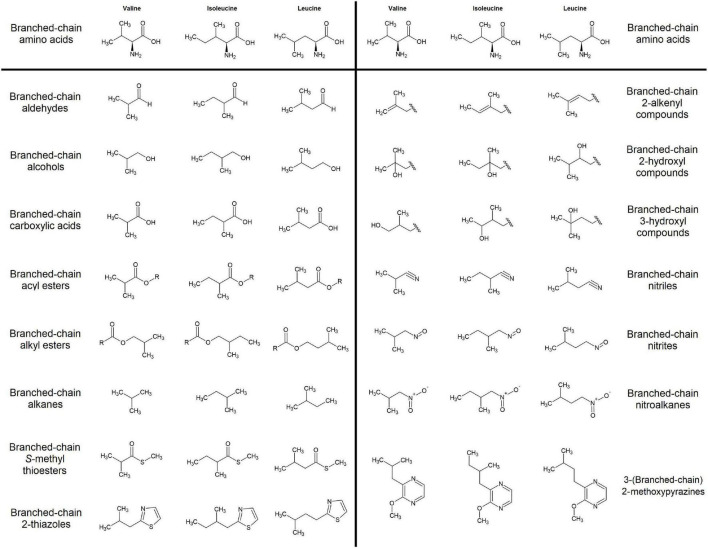
Categories of branched-chain volatile compounds detected in edible fruits. At least one volatile from each family was reported in at least one of the 175 fruit volatile studies examined in this review (see Supplementary Table 1).

Branched chain volatiles are defined as those volatile organic compounds that contain a branched-chain functional group structurally similar to those of the branched-chain amino acids- valine, leucine, and isoleucine (Figure 1). Due to this structural similarity BCVs were theorized to derive from the metabolism of branched-chain amino acids (BCAAs), a hypothesis supported by numerous feeding experiments (Tressl and Drawert, 1973; Hansen and Poll, 1993; Rowan et al., 1996; Wyllie and Fellman, 2000; Pérez et al., 2002; Matich and Rowan, 2007; Gonda et al., 2010). Yet while thorough work has been done elucidating the biosynthesis and metabolism of branched-chain amino acids in plants (Singh and Shaner, 1995; Taylor et al., 2004; Binder et al., 2007; Binder, 2010; Xing and Last, 2017), the precise enzymatic mechanisms by which BCAA metabolism diverges into BCV biosynthesis remain relatively understudied. Furthermore, recent experimental evidence indicates that under certain circumstances BCV production may occur independently of normal BCAA metabolism (Sugimoto, 2011; Kochevenko et al., 2012; Sugimoto et al., 2021). A more complete understanding of BCV biosynthesis in plants would be of great importance to researchers working to better understand the molecular basis of fruit flavor, and in particular to those who desire to apply such knowledge to the development of novel fruit varieties with improved flavor traits.

This review seeks to collate and evaluate published research regarding the biosynthesis of branched chain volatiles in plants, with an emphasis on these processes as they might occur in fruit crops. While BCV biosynthesis has been touched upon in other reviews on the subject of plant volatiles (Dudareva et al., 2006, 2013; El Hadi et al., 2013), a comprehensive exploration of the state of current knowledge concerning the molecular basis of BCV biosynthesis in plants has yet to be published.

## Branched-Chain Volatiles in Fruits

Branched-chain volatiles are ubiquitous components of fruit aroma volatile profiles. An examination of research literature concerning fruit volatile profiles found that 127 unique branched-chain volatile compounds were identified in 106 distinct types of edible fruit across 175 published studies (Table 1). A list of all BCVs reported for a particular fruit by these studies is given in Supplementary Table 1. These fruits include representatives from 22 individual taxonomic orders spanning monocots, eudicots, and magnoliids, suggesting that the production of branched-chain volatiles in fruit tissue is a characteristic with widespread evolutionary utility. It has been proposed that since many fruit volatiles are derived from nutritionally significant compounds, their production in fruit tissue might be a means of signaling nutritional value to seed-dispersing frugivores (Goff and Klee, 2006). Because animals are incapable of biosynthesizing branched-chain amino acids (Hou and Wu, 2018), volatile cues enabling the identification of food sources rich in these compounds could serve as powerful attractants to animal seed-dispersers. This may be one explanation for the ubiquity of branched-chain volatiles in fruits from plants across many divergent evolutionary lineages, though much more research is needed to firmly establish this hypothesis. Conversely BCVs may also find use as defense agents against herbivorous predators, an application associated with certain nitrogen-containing branched-chain VOCs (Irmisch et al., 2013, 2014).

**TABLE 1:** List of all branched-chain volatile compounds detected in 106 edible fruits across 175 published studies of fruit volatile content.

Compound name	CAS #	Molecular weight	Number of fruits identified in
***Alcohols***			
2-methyl-1-propanol	78-83-1	74.12	34
2-methyl-1-butanol	137-32-6	88.15	32
3-methyl-1-butanol	123-51-3	88.15	64
***Aldehydes***			
2-methylpropanal	78-84-2	72.11	12
2-methylbutanal	96-17-3	86.13	20
3-methylbutanal	590-86-3	86.13	31
***Alkanes***			
2-methylbutane	78-78-4	72.15	1
***Carboxylic acids***			
2-methylpropanoic acid	79-31-2	88.11	13
2-methylbut-2-enoic acid	13201-46-2	100.12	1
2-methylbutanoic acid	116-53-0	102.13	26
3-methylbutanoic acid	503-74-2	102.13	26
2-methyl-3-hydroxypropanoic acid	2068-83-9	104.1	1
***Esters***			
Methyl 2-methylprop-2-enoate	80-62-6	100.12	1
Methyl 2-methylpropanoate	547-63-7	102.13	4
Methyl 2-methylbut-2-enoate	6622-76-0	114.14	1
Methyl 3-methylbut-2-enoate	924-50-5	114.14	2
Ethyl 2-methylprop-2-enoate	97-63-2	114.14	1
2-methylpropyl acetate	110-19-0	116.16	29
Methyl 2-methylbutanoate	868-57-5	116.16	24
Methyl 3-methylbutanoate	556-24-1	116.16	13
Ethyl 2-methylpropanoate	97-62-1	116.16	29
Ethyl 2-methylbut-2-enoate	5837-78-5	128.17	12
Ethyl 3-methylbut-2-enoate	638-10-8	128.17	1
Propyl 2-methylprop-2-enoate	2210-28-8	128.17	1
2-methylpropyl propanoate	540-42-1	130.18	1
2-methylbutyl acetate	624-41-9	130.18	18
3-methylbutyl acetate	123-92-2	130.18	52
Ethyl 2-methylbutanoate	7452-79-1	130.18	49
Ethyl 3-methylbutanoate	108-64-5	130.18	27
Propyl 2-methylpropanoate	644-49-5	130.18	1
1-methylethyl 2-methylpropanoate	617-50-5	130.18	2
Methyl 2-hydroxy-2-methylbutanoate	32793-34-3	132.16	2
Methyl 2-hydroxy-3-methylbutanoate	17417-00-4	132.16	7
Methyl 3-hydroxy-3-methylbutanoate	6149-45-7	132.16	4
Propyl 2-methylbut-2-enoate	61692-83-9	142.2	1
2-methylpropyl butanoate	539-90-2	144.12	13
2-methylpropyl 2-methylpropanoate	97-85-8	144.21	4
2-methylbutyl propanoate	2438-20-2	144.21	2
3-methylbutyl propanoate	105-68-0	144.21	1
Propyl 2-methylbutanoate	37064-20-3	144.21	6
Propyl 3-methylbutanoate	557-00-6	144.21	5
Butyl 2-methylpropanoate	97-87-0	144.21	6
2-methylpropyl 2-hydroxypropanoate	585-24-0	146.18	1
Ethyl 2-hydroxy-2-methylbutanoate	77-70-3	146.18	2
Ethyl 2-hydroxy-3-methylbutanoate	2441-06-7	146.18	2
Ethyl 3-hydroxy-3-methylbutanoate	18267-36-2	146.18	4
2-methylpropyl 2-methylbut-2-enoate	7779-81-9	156.22	4
3-methylbut-3-enyl 2-methylpropanoate	76649-23-5	156.22	1
Butyl 2-methylbut-2-enoate	7785-66-2	156.23	2
2-methylpropyl 2-methylbutanoate	2445-67-2	158.24	3
2-methylpropyl 3-methylbutanoate	589-59-3	158.24	5
2-methylbutyl butanoate	51115-64-1	158.24	5
2-methylbutyl 2-methylpropanoate	2445-69-4	158.24	1
3-methylbutyl butanoate	106-27-4	158.24	14
3-methylbutyl 2-methylpropanoate	2050-01-3	158.24	6
Butyl 2-methylbutanoate	15706-73-7	158.24	8
Butyl 3-methylbutanoate	109-19-3	158.24	9
Pentyl 2-methylpropanoate	2445-72-9	158.24	4
3-methylbut-3-enyl 2-methylbut-2-enoate	83783-87-3	168.23	1
3-methylbut-3-enyl 3-methylbutanoate	54410-94-5	170.25	2
Hexyl 2-methylprop-2-enoate	142-09-6	170.25	1
Pentan-2-yl 3-methylbut-2-enoate	150462-84-3	170.25	1
(Z)-3-hexenyl 2-methylpropanoate	41519-23-7	170.25	6
2-methylpropyl hexanoate	105-79-3	172.26	10
2-methylbutyl 2-methylbutanoate	2445-78-5	172.26	2
2-methylbutyl 3-methylbutanoate	2445-77-4	172.26	3
3-methylbutyl pentanoate	2050-09-1	172.26	1
3-methylbutyl 2-methylbutanoate	27625-35-0	172.26	2
3-methylbutyl 3-methylbutanoate	659-70-1	172.26	6
Pentyl 2-methylbutanoate	68039-26-9	172.26	1
Pentyl 3-methylbutanoate	25415-62-7	172.26	2
Hexyl 2-methylpropanoate	2349-07-7	172.26	10
Pentan-2-yl 3-methylbutanoate	117421-34-8	172.27	1
2-methylpropyl benzoate	120-50-3	178.23	2
3-methylbutyl (E)-2-hexenoate	72928-34-8	184.28	1
Hexan-2-yl 3-methylbut-2-enoate	N/A	184.28	1
(E)-2-hexenyl 2-methylbutanoate	94089-01-7	184.28	1
(Z)-3-hexenyl 2-methylbutanoate	53398-85-9	184.28	4
(Z)-3-hexenyl 3-methylbutanoate	35154-45-1	184.28	4
2-methylbutyl hexanoate	2601-13-0	186.29	3
3-methylbutyl hexanoate	2198-61-0	186.29	16
Hexyl 2-methylbutanoate	10032-15-2	186.29	13
Hexyl 3-methylbutanoate	10032-13-0	186.29	11
Heptyl 2-methylpropanoate	2349-13-5	186.29	1
Benzyl 2-methylbut-2-enoate	37526-88-8	190.24	1
2-methylpropyl phenylacetate	102-13-6	192.25	1
2-methylbutyl benzoate	52513-03-8	192.25	1
3-methylbutyl benzoate	94-46-2	192.25	1
Benzyl 3-methylbutanoate	103-38-8	192.25	4
2-phenylethyl 2-methylpropanoate	103-48-0	192.25	2
(E)-4-hepten-2-yl 3-methylbutanoate	N/A	198.3	1
(Z)-4-hepten-2-yl 3-methylbutanoate	N/A	198.3	1
2-methylpropyl octanoate	5461-06-3	200.32	4
Octyl 2-methylpropanoate	109-15-9	200.32	4
2-phenylethyl 2-methylbut-2-enoate	55719-85-2	204.26	1
2-phenylethyl 2-methylbutanoate	24817-51-4	206.28	1
2-phenylethyl 3-methylbutanoate	140-26-1	206.28	3
3-phenylpropyl 2-methylpropanoate	103-58-2	206.28	1
3-methylbutyl 2-aminobenzoate	28457-05-8	207.27	1
2-phenoxyethyl 2-methylpropanoate	103-60-6	208.26	2
(E)-4-octenyl 3-methylbutanoate	N/A	212.33	1
(Z)-4-octenyl 3-methylbutanoate	N/A	212.33	1
(Z)-5-octenyl 3-methylbutanoate	N/A	212.33	1
2-methylbutyl octanoate	67121-39-5	214.34	2
3-methylbutyl octanoate	2035-99-6	214.34	8
Octyl 2-methylbutanoate	29811-50-5	214.34	1
Octyl 3-methylbutanoate	7786-58-5	214.34	2
Cinnamyl 3-methylbutanoate	140-27-2	218.29	1
3-phenylpropyl 3-methylbutanoate	5452-07-3	220.31	1
Neryl 2-methylpropanoate	2345-24-6	224.34	2
2-methylpropyl decanoate	30673-38-2	228.37	1
Decyl 2-methylpropanoate	5454-22-8	228.37	1
Geranyl 3-methylbutanoate	109-20-6	238.37	2
(Z)-4-decenyl 3-methylbutanoate	N/A	240.38	1
3-methylbutyl decanoate	2306-91-4	242.4	2
2-methylpropyl dodecanoate	37811-72-6	256.42	1
3-methylbutyl dodecanoate	6309-51-9	270.45	1
2-methylpropyl hexadecanoate	110-34-9	312.54	1
3-methylbutyl hexadecanoate	81974-61-0	326.56	1
2-methylpropyl octadecanoate	646-13-9	340.58	1
***Nitriles***			
2-methylpropylnitrile	78-82-0	69.11	1
3-methylbutylnitrile	625-28-5	83.13	1
***Nitrites***			
3-methylbutylnitrite	110-46-3	117.15	1
***Nitroalkanes***			
3-methyl-1-nitrobutane	627-67-8	117.15	1
***Pyrazines***			
2-(2-methylpropyl)-3-methoxypyrazine	24683-00-9	166.22	6
***Thiazoles***			
2-(2-methylpropyl)-thiazole	18640-74-9	141.23	1
***Thioesters***			
*S*-methyl 3-methylbutanethioate	23747-45-7	132.22	1

Compounds are identified by both name and CAS registry number and are grouped according to the structural categories given in Figure 1. An absence of a CAS registry number for a compound is indicated by “N/A.” Within structural categories, compounds are listed in descending order according to molecular weight. Also provided is the number of distinct edible fruits each compound has been reported in by the sources cited throughout this. For a complete list of which branched-chain volatile compounds were detected in a given fruit along with the corresponding reporting studies, please refer to Supplementary Table 1.

An overwhelming majority of the branched-chain volatiles identified in fruits are classified as volatile esters. Out of the 127 distinct BCVs identified in Table 1, 108 are esters. Many of these are conjugate esters derived from a branched-chain structure attached to a compound from a completely different biosynthetic origin. In this way, branched-chain volatiles incorporating structures derived from terpenoid, phenylpropanoid, and oxylipin metabolism are formed- allowing for massive diversity in the number of possible volatiles containing a branched-chain structure. The remaining non-ester BCVs observed in Table 1 include branched-chain alcohols, aldehydes, alkanes, and carboxylic acids. Several BCVs containing more unusual functional groups are also listed, including compounds with nitrile, nitrite, nitro, and thioester functional groups.

Branched-chain volatiles have been recognized as being crucially responsible for the distinctive flavor and aroma properties of many commercially important fruits such as apple, banana, melon, and pineapple, among others (Wyllie et al., 1995; Plotto, 1998; Dixon and Hewett, 2000; Boudhrioua et al., 2003; Tokitomo et al., 2005; Wendakoon et al., 2006). In terms of effect on human sensory perception it has been widely reported that branched-chain volatile esters tend to impart characteristic “fruity” aroma notes, while non-ester branched-chain volatiles induce a broader range of olfactory sensations (Schwab et al., 2008; Sugimoto, 2011; El Hadi et al., 2013; Lytra et al., 2014). Fruity aroma is a characteristic shared with several other non-branched-chain volatile compounds, particularly short- and medium- length straight-chain esters (Schwab et al., 2008; El Hadi et al., 2013). However, it has been observed that ester compounds containing the branched-chain moiety have significantly lower odor thresholds than the corresponding straight-chain counterparts (Takeoka et al., 1995), indicating that BCVs may be more potent stimulators of the “fruity” olfactory sensation. It is important to communicate that the “fruity” aroma notes imparted by BCVs are not always associated with positive consumer responses, and in some fruits are associated with decreased consumer ratings (Goulet et al., 2012).

## Current Knowledge Regarding Branched-Chain Volatile Biosynthesis

**FIGURE 2:**
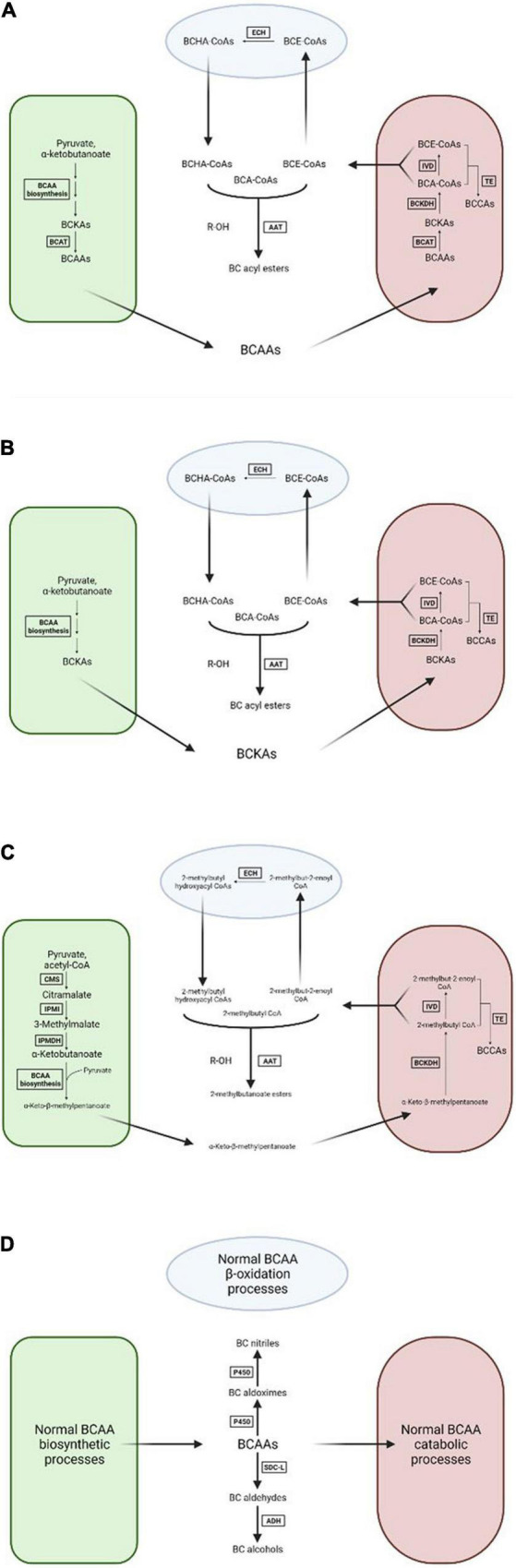
Visual diagrams of the four hypotheses concerning branched-chain volatile biosynthesis in plant cells. **(A)** Mitochondrial catabolism of branched-chain amino acids. **(B)** *De novo* branched-chain α-ketoacid biosynthesis followed by mitochondrial catabolism. **(C)** Production of 2-methylbutyl volatiles by citramalate synthase. **(D)** Direct transformation of branched-chain amino acids. BCAA, branched-chain amino acid; BCKA, branched-chain α-ketoacid; BCAT, branched-chain amino acid aminotransferase; BCKDH, branched-chain α-ketoacid dehydrogenase complex; IVD, isovaleryl-CoA dehydrogenase; TE, thioesterase; ECH, enoyl-CoA hydratase; AAT, alcohol acyltransferase; CMS, citramalate synthase; IPMI, isopropylmalate isomerase; IPMDH, isopropylmalate dehydrogenase; P450, cytochrome P450 enzyme; SDC-L, serine-decarboxylase like enzyme; ADH, alcohol dehydrogenase. Green compartment represents the chloroplast, maroon compartment the mitochondrion, light blue compartment the peroxisome, and white background the cytosol. Arrows with faded ends indicate cross-membrane transport.

Because of the importance of branched-chain volatiles to the characteristic flavors of several important fruit crops much research has been done in trying to understand the general biosynthesis pathways that lead to BCV production, though this area remains relatively understudied when compared to the biosynthetic processes that produce other classes of important fruit volatiles such as terpenoids and oxylipins. The body of published experimental evidence regarding this topic points to four possible hypotheses regarding BCV biosynthesis (Figure 2), none of which are mutually exclusive with any of the others. These hypotheses are summarized below.

### Mitochondrial Catabolism of Branched-Chain Amino Acids

The oldest and most studied of the four possibilities, this hypothesis posits that BCVs are generated through the catabolic degradation of BCAAs in mitochondria. It has long been known that plants possess the capability to break down excess BCAAs into the energy-rich metabolites acetyl-CoA and propionyl-CoA and that several of the key enzymes involved in this process are localized to mitochondria (Binder, 2010; Hildebrandt et al., 2015). An excellent overview of this pathway as hypothesized to occur in plants is provided in Binder (2010). Briefly, this process begins with the deamination of BCAAs by branched-chain aminotransferases (BCATs) followed by decarboxylation of the resulting branched-chain α-ketoacids (BCKAs) through the branched-chain α-ketoacid dehydrogenase complex (BCKDH). This results in the formation of various branched-chain acyl-CoAs (BCA-CoAs), which in turn are reduced at the α-carbon through the action of isovaleryl-CoA dehydrogenases or functionally similar enzymes. These reduced BCA-CoAs then undergo β-oxidation into acetyl-CoA and propionyl-CoA, though whether these later stages occur in mitochondria or in peroxisomes remains unresolved (Graham and Eastmond, 2002; Kaur et al., 2009).

Since numerous volatile esters are known to be biosynthesized in fruit tissue by alcohol acyltransferase (AAT) enzymes (Beekwilder et al., 2004; El-Sharkawy et al., 2005; Souleyre et al., 2005; Günther et al., 2011; Goulet et al., 2015) and because AATs require an alcohol and an acyl-CoA as substrates, the branched-chain acyl-CoAs produced by mitochondrial BCAA catabolism would naturally be considered likely candidates for conversion to volatile branched-chain acyl esters. Feeding and isotope labeling experiments conducted in apple, banana, and strawberry provide strong evidence that BCAAs can be directly converted to volatile branched-chain acyl esters in fruit tissue (Tressl and Drawert, 1973; Rowan et al., 1996; Wyllie and Fellman, 2000; Pérez et al., 2002). Furthermore, the detection in several fruits of volatile branched-chain acyl esters with double bonds and hydroxyl groups in positions necessary for β-oxidation to proceed indicate that intermediaries of late-stage BCAA catabolism are actively incorporated into volatile fruit compounds. And it has also been demonstrated in hops that a mitochondrial thioesterase is capable of cleaving BCA-CoAs into branched-chain carboxylic acids (Xu et al., 2013), important aroma volatiles detected in many kinds of fruit (Supplementary Table 1). Notably, it was found that when apple and banana fruit tissue was fed with deuterium-labeled BCAAs, deuterated branched-chain carboxylic acid production was observed (Tressl and Drawert, 1973; Rowan et al., 1996). Taken together, these lines of evidence strongly support the hypothesis that fruit BCVs are formed through catabolic breakdown of BCAAs.

However, several difficulties remaining with this hypothesis indicate that it may not be generally applicable across all fruits. Evidence from tomato seems to show that BCAA catabolism may not be primarily responsible for the formation of BCVs in that fruit; when disks of tomato fruit tissue were fed elevated levels of BCAAs, no significant measurable increase in BCV quantity was detected as compared to control (Kochevenko et al., 2012). Furthermore, overexpression of tomato BCAT genes did not result in plants yielding fruits that produced increased levels of BCVs (Kochevenko et al., 2012). Since, BCAT enzymes catalyze the first step in BCAA catabolic breakdown, overexpression of the genes encoding these proteins should theoretically result in higher levels of BCAAs entering the degradation pathway which in turn should yield more substrates for conversion into greater amounts of BCVs- results not observed in the tomato BCAT overexpression experiments. Other difficulties with the BCAA catabolism hypothesis deal with the issue of cellular regulation of BCAA metabolism: since it is known that under ordinary conditions the biosynthesis of BCAAs is tightly regulated through feedback inhibition (Binder, 2010; Galili et al., 2016; Xing and Last, 2017), it must be explained how some fruits can overcome these regulatory barriers to produce the great amounts of BCAAs and corresponding breakdown products needed to support the biosynthesis of massive quantities of BCVs observed in these fruits. By itself, the BCAA catabolism hypothesis is incapable of accounting for this. These difficulties have led to the proposal and investigation of other hypotheses regarding possible alternate routes for BCV biosynthesis.

### *De novo* Branched-Chain α-Ketoacid Biosynthesis Followed by Mitochondrial Catabolism

Based primarily off of research done in tomato, this hypothesis posits that BCAAs are not the main source of carbon used for biosynthesis of fruit BCVs but that instead it is branched-chain α-ketoacids that fill this role. In this hypothesis, the biosynthesis of BCAAs proceeds as normal up until the last step, where instead of conversion to BCAAs by BCAT enzymes branched-chain α-ketoacids produced so far are directly exported to the mitochondria for BCKDH-mediated catabolism into BCVs and associated precursors. This hypothesis has several explanatory advantages. For one, it explains the findings in tomato that feeding excess BCAAs to tomato fruit tissue does not result in elevated BCV volatile production, while feeding excess BCKAs does (Kochevenko et al., 2012). Another advantage is that this hypothesis manages to bypass BCAA-mediated feedback inhibition of upstream biosynthesis enzymes, though inhibition by non-BCAA precursors generated by this pathway may still occur depending on the sensitivities of these enzymes to those compounds.

Much more experimental evidence is needed to conclude whether or not this hypothesized pathway is involved in BCV biosynthesis in certain fruits. In particular, labeling experiments where deuterated BCKAs are fed to fruit tissue and deuterated BCVs are observed but not deuterated BCAAs would provide robust evidence for the validity of this hypothesis. Supporting evidence could include an observation that BCAT transcripts become drastically reduced in fruit tissue as compared to the other upstream BCAA biosynthesis enzymes. These *in vitro* assays would show that BCKAs do not cause feedback inhibition of the upstream biosynthesis enzyme isoforms present in fruit, or the discovery and characterization of a chloroplast transporter expressed in fruit tissue that preferentially exports BCKAs before they can be converted to BCAAs by chloroplastic BCATs.

It is important to note that this hypothesis may not be mutually exclusive with the BCAA catabolism hypothesis: it may be possible that fruit cells produce elevated levels of BCKAs while also simultaneously catabolizing BCAAs into volatiles. In fact, doing both might actually increase the quantity of carbon shunted into mitochondrial BCKDH-mediated volatile precursor production: catabolizing BCAAs would reduce inhibitory pressure on key BCAA biosynthesis enzymes which would then be free to produce greater quantities of BCKAs that would be directly used to support BCV production. Further investigation is needed to determine if such a scenario indeed occurs in fruit tissue in nature and if so, what quantifiable impact each pathway has on overall BCV production.

### Production of 2-Methylbutyl Volatiles by Citramalate Synthase

Work in apple has revealed a third possible route for the biosynthesis of BCVs. Unlike the first two hypothesized pathways, this route does not involve the early steps of classical plant BCAA biosynthesis but instead relies on the enzyme citramalate synthase (CMS), an enzyme previously known to be involved in BCAA biosynthesis only in bacteria (Sugimoto, 2011; Sugimoto et al., 2021). Briefly, in this pathway citramalate synthase condenses one molecule of pyruvate with one molecule of acetyl-CoA to form the dicarboxylic acid citramalate, a molecule of which is then converted to 3-methylmalate by the action of isopropylmalate isomerase (IPMI) enzymes. This compound is then acted upon by isopropylmalate dehydrogenase (IPMDH) enzymes to yield α-ketobutanoate, a known precursor of isoleucine biosynthesis via the established plant pathway. Biosynthesis to α-keto-β-methylpentanoate and isoleucine then proceeds along the conventional plant BCAA biosynthesis pathway. Direct experimental evidence for the activity of this pathway in apple has been obtained through the use of ^13^C-labeled acetate feeding and *in vitro* biochemical characterization of CMS and IPMI enzymes expressed in apple fruit tissue (Sugimoto, 2011; Sugimoto et al., 2021). Supporting evidence in the form of measured increases in levels of citramalate and CMS transcripts as apple ripening progressed was also obtained in the same studies.

The primary explanatory advantage of this hypothesis is that it enables the production of massive quantities of 2-methylbutyl volatile compounds in fruit without being subject to the feedback inhibition that large concentrations of isoleucine exert on threonine deaminase (TD), an enzyme that catalyzes the first committed step in isoleucine biosynthesis (Sidorov et al., 1981; Singh and Shaner, 1995; Binder, 2010; Sugimoto et al., 2021). Furthermore, additional evidence in apple indicates that fruit CMS enzymes may also play a role in biosynthesizing straight-chain esters, a class of compound commonly found at high levels in several fruits including apple (Plotto, 1998; El Hadi et al., 2013; Liu et al., 2021; Sugimoto et al., 2021). Nevertheless, this hypothesis alone suffers from a serious limitation: it is unable to account for the high levels of 3-methylbutyl volatiles observed in several fruits as well as elevated levels of 2-methylpropyl volatiles observed in others. Therefore, other hypotheses must be deferred to when considering the biosynthetic mechanisms underlying the formation of 3-methylbutyl and 2-methylpropyl fruit volatiles. The fact that 3-methylbutyl and 2-methylpropyl compounds have been detected in apple alongside high levels of 2-methylbutyl volatiles (Qin et al., 2017; Liu et al., 2021) seems to indicate that at least two separate and distinct BCV biosynthesis pathways can be active at the same time in this fruit.

### Direct Transformation of Branched-Chain Amino Acids

The last possible mechanism of BCV biosynthesis is direct conversion of BCAAs into volatile compounds or immediate precursors. Rather than undergoing several steps of degradation through the established BCAA catabolism process before being incorporated into BCVs, this hypothesis proposes that some volatiles are directly transformed through enzymatic action into BCVs or the immediate precursors of such. Evidence from alfalfa and chickpea indicates that plants form branched-chain aldehydes and alcohols through this pathway: in both species, cDNAs for serine decarboxylase-like (SDC-L) enzymes were found that when heterologously expressed in bacteria produced enzymes capable of directly forming branched-chain aldehydes from branched-chain amino acids (Torrens-Spence et al., 2014). Branched-chain alcohols could then be formed through the action of alcohol dehydrogenase (ADH) enzymes acting on these branched-chain aldehydes. Similarly, work done in poplar demonstrated that branched-chain nitrile volatiles can be biosynthesized from BCAAs through the action of two cytochrome P450 enzymes, one that converts BCAAs into branched-chain aldoximes and another that subsequently converts the branched-chain aldoximes into branched-chain nitriles (Irmisch et al., 2013, 2014). Presumably, this pathway may also be involved in the biosynthesis of other nitrogen-containing BCVs reported from various fruits as it does not involve the loss of the original amino acid’s nitrogen atom but permits its refunctionalization into another chemical moiety. Indeed, evidence from grape indicates that branched-chain 2-methoxypyrazine volatiles are formed in just such a manner (Lei et al., 2019). Experiments involving feeding of ^15^N-labeled BCAAs to tissue from fruits known to produce nitrogenous BCVs would be one way to empirically demonstrate such a role for this pathway. The biosynthesis of both types of branched-chain volatile compound have been shown to be accomplished without the involvement of the mitochondrial BCKDH enzyme complex, a key feature of the previous three hypotheses.

**FIGURE 3:**
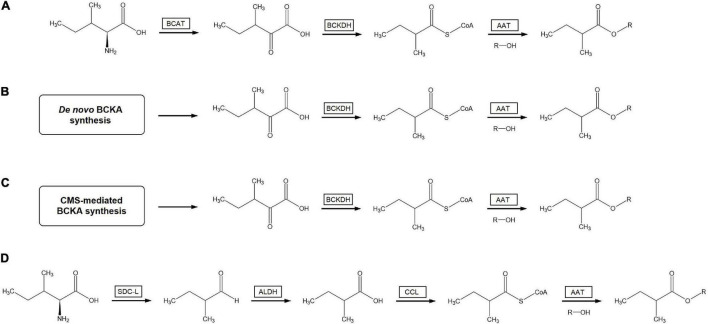
Possible biosynthesis routes to volatile branched-chain acyl esters. **(A)** Mitochondrial BCAT- and BCKDH-mediated catabolism of free branched-chain amino acids to branched-chain acyl-CoAs followed by esterification with alcohols by AAT enzymes. **(B)** *De novo* chloroplast synthesis of branched-chain α-ketoacids followed by BCKDH-mediated catabolism to branched-chain acyl-CoAs and subsequent AAT-mediated esterification with alcohols. **(C)** CMS-initiated synthesis of α-keto-β-methylpentanoate followed by BCKDH-mediated catabolism to 2-methylbutyl acyl-CoA and subsequent AAT-mediated esterification with alcohols to form 2-methylbutanoate esters. **(D)** Branched-chain aldehyde synthesis from free branched-chain amino acids via SDC-L, followed by conversion of branched-chain aldehydes to branched-chain carboxylic acids through ALDH enzymes, followed by activation to branched-chain acyl-CoAs by CCL enzymes and subsequent condensation with alcohols via AAT to generate branched-chain acyl esters. The first, second, and fourth routes are capable of generating acyl esters with all three branched-chain structures, while the third route can only generate 2-methylbutanoate esters. The four pathways are illustrated yielding 2-methylbutanoate esters from appropriate precursors for ease of comparison. BCAT, branched-chain amino acid aminotransferase; BCKDH, branched-chain α-ketoacid dehydrogenase complex; AAT, alcohol acyltransferase; BCKA, branched-chain α-ketoacids; SDC-L, serine-decarboxylase like enzyme; ALDH, aldehyde dehydrogenase; CCL, carboxyl-CoA ligase.

It is interesting to note that this pathway can theoretically provide a possible route for the biosynthesis of branched-chain acyl esters that also does not rely on the activity of the mitochondrial BCKDH complex (Figure 3). In this proposed route, branched-chain aldehydes are generated by a serine decarboxylase-like enzyme as per that characterized by Torrens-Spence et al. (2014). These branched-chain aldehydes would then be converted to branched-chain carboxylic acids by the action of aldehyde dehydrogenases (ALDHs), enzymes known to be found in the genomes of many plants (Brocker et al., 2013; Tola et al., 2021). The resulting branched-chain carboxylic acids could then be turns into the corresponding branched-chain acyl-CoAs by carboxyl-CoA ligases (CCLs), as was demonstrated to occur in hops (Xu et al., 2013). This route is fully compatible with existing data from feeding and labeling experiments that demonstrate the conversion of BCAAs into branched-chain acyl esters. In addition, given that neither of the known BCV-forming SDC-like enzymes are predicted to localize to mitochondria (Torrens-Spence et al., 2014) and that several plant ALDHs are known to be localized to the cytosol (Končitíková et al., 2015; Tola et al., 2021) along with CCLs shown to activate branched-chain substrates (Xu et al., 2013), this proposed pathway solves the difficulty of explaining how BCA-CoAs produced in the mitochondria can cross the mitochondrial membrane into the cytosol where plant AAT enzymes have been shown to be localized (Noichinda et al., 1999a; Zhang et al., 2019b). Furthermore, since prior research indicates that mitochondrial BCKDH-mediated BCAA catabolism is tightly regulated by a variety of factors in plants (Fujiki et al., 2001; Peng et al., 2015) a BCA-CoA production pathway not involving the BCKDH complex would not need to overcome in-built regulatory hurdles to generate large quantities of BCA-CoAs. Several difficulties are still apparent with this hypothetical route to branched-chain acyl esters: the accumulation of high levels of aldehyde compounds is known to be toxic to cells, competition for aldehyde substrates with the ADH enzymes known to produce branched-chain alcohols, and the simple fact that no direct evidence of this alternate route has yet been empirically demonstrated. Experimental evidence such as deuterium-labeled branched-chain aldehydes yielding branched-chain acyl esters with deuterated acyl moieties or the characterization of fruit ALDH enzymes capable of forming branched-chain carboxylic acids from branched-chain aldehydes would go a long way to establish the viability of this proposed pathway to branched-chain acyl ester biosynthesis.

## Unresolved Questions Concerning Branched-Chain Volatile Biosynthesis

Despite significant progress in understanding the molecular correlates of BCV biosynthesis in plants, much remains to be uncovered. In comparison, the metabolic bases of the biosynthesis of other important classes of fruit volatile such as the terpenoid, oxylipin, and phenylpropanoid families have been quite thoroughly characterized. For a similar level of understanding to be achieved for branched-chain volatiles, several important questions need to be resolved. A few of these unresolved questions regarding BCV biosynthesis most relevant to fruit volatile metabolism are explored below:

### How Branched-Chain α-Ketoacid Dehydrogenase Complex-Mediated Branched-Chain Amino Acid Catabolism Is Regulated in Fruit Tissue

As has been discussed in previous sections of this review, branched-chain acyl esters have been shown to be among the most abundant BCVs detected in several important fruits and have also been demonstrated to be key components of many characteristic fruit flavors. The characterization of numerous fruit alcohol acyltransferase enzymes indicates that these volatiles are most likely formed through the condensation of alcohols with branched-chain acyl-CoAs (Beekwilder et al., 2004; El-Sharkawy et al., 2005; Souleyre et al., 2005; Günther et al., 2011). In three of the four hypotheses regarding BCV biosynthesis previously explored in this review, the mitochondrial BCKDH enzyme complex plays the critical role in generating the BCA-CoAs necessary for branched-chain acyl ester biosynthesis. Evidence in plants indicates that the activity of this complex is regulated by several factors (Fujiki et al., 2001; Peng et al., 2015), while work done on the far more studied mammalian BCKDH complex has identified several molecules that directly modulate BCKDH activity. These include a kinase which directly suppresses BCKDH activity and can itself be inhibited by branched-chain α-ketoacids (Paxton and Harris, 1984; Harris et al., 1986, 1997), phosphatase enzymes that reverse the effects of the kinase and promote activity of the BCKDH complex (Lu et al., 2009; Zhou et al., 2012) and even BCA-CoAs themselves, which have been shown to suppress BCKDH activity *in vitro* (Parker and Randle, 1978).

The overall question relevant to researchers working in the field of fruit aroma volatile metabolism is what role if any do these BCKDH regulatory mechanisms play in the generation of branched-chain acyl esters? If regulatory mechanisms that directly or indirectly suppress BCKDH activity in plants are identified, how are they overcome in fruit tissues to produce the great quantities of BCA-CoAs needed to support large-scale branched-chain acyl ester biosynthesis as observed in fruits such as apple? If plant homologs of the mammalian BCKDH kinase and phosphatase enzymes are found, are their expression levels in fruit negatively or positively correlated with branched-chain acyl ester content? Are subunits of the plant BCKDH complex inhibited by high levels of BCA-CoAs as was demonstrated to occur in the mammalian complex (Parker and Randle, 1978)? The answers to these questions and related ones would shed great insight into an important aspect of fruit BCV production and may even offer potential avenues for altering the BCV content of fruits by manipulating or even bypassing the underlying regulatory mechanisms governing branched-chain acyl ester precursor biosynthesis.

### What Biosynthetic Processes Form Sulfur-Containing Branched-Chain Volatiles

In this review’s survey of 175 published studies of the volatile profiles of various fruits only two sulfur-containing BCVs, 2-(2-methylpropyl)-thiazole and *S*-methyl 3-methylbutanethioate, were found (Table 1). Compared with the 125 other branched-chain compounds reported, this would seem to indicate that sulfur-containing BCVs are of little importance to the study of fruit volatile metabolism. Nevertheless, the detection of one of these compounds [2-(2-methylpropyl)-thiazole] in the economically significant tomato fruit at concentrations far above the minimum odor threshold (Baldwin et al., 2000; Tieman et al., 2012) as well as the importance of sulfur-containing volatile compounds in general to the flavor of numerous tropical fruits (Engel, 1999; Cannon and Ho, 2018) justifies a closer look at the biosynthesis of sulfur-containing BCVs.

The compound 2-(2-methylpropyl)-thiazole, hereafter referred to as 2-isobutylthiazole, is a known enhancer of tomato flavor that finds frequent use in the preparation of artificial condiments (Kazeniac and Hall, 1972; Christiansen et al., 2011). It has been detected in ripe tomato fruits by several studies (Baldwin et al., 2000; Tikunov et al., 2005; Tieman et al., 2012). Very little is known about the biosynthesis of this compound (Paolo et al., 2018). Hierarchically clustered metabolite data from several tomato introgressed lines characterized for volatile content seems to indicate a branched-chain amino acid origin for this compound (Mathieu et al., 2009). It is the precise nature of how this biosynthetic process occurs that remains totally unknown. That plants possess the capacity to biosynthesize the thiazole moiety is well known from studies examining plant biosynthesis of thiamine (Belanger et al., 1995; Goyer, 2010). However, from a biochemistry perspective it is difficult to see how this established pathway could incorporate a branched-chain amino acid. It is far more likely that the biosynthesis of this volatile occurs via a novel mechanism that uses a leucine molecule as a starting point and source for the thiazole’s nitrogen. Feeding ^15^N-labeled leucine to portions of tomato fruit tissue and examining any generated 2-isobutylthiazole molecules for that radioisotope could confirm or refute that assertion. Far more difficult would be accounting for the sulfur component of the thiazole ring and the two carbons at the four- and five-positions. While experiments using radiolabeled cysteine or methionine could yield some insight into the thiazole ring’s origin, ultimately it may be more feasible to use the abundant genetic resources available for tomato to track down potential biosynthesis enzymes using fine mapping of quantitative trait loci robustly associated with variable levels of this compound.

**FIGURE 4:**
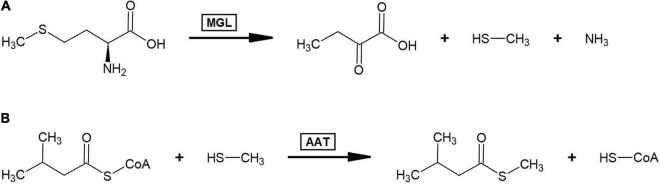
Proposed biosynthesis pathway for *S*-methyl branched-chain thioester volatiles. **(A)** Breakdown of L-methionine to α-ketobutanoate, methanethiol, and ammonia by the action of L-methionine-γ–lyase (MGL). **(B)** Formation of *S*-methyl branched-chain thioesters via alcohol acyltransferase (AAT) mediated condensation of methanethiol with branched-chain acyl-CoAs. This panel illustrates this process occurring with 3-methylbutyl-CoA and yielding *S*-methyl 3-methylbutanethioate since that was the only *S*-methyl branched-chain thioester volatile identified across the 175 fruit volatile studies examined in this review; however, this process could theoretically yield *S*-methyl 2-methylpropanethioate and *S*-methyl 2-methylbutanethioate from 2-methylpropyl-CoA and 2-methylbutyl-CoA, respectively.

The other sulfur-containing BCV identified is the compound *S*-methyl 3-methylbutanethioate, isolated from cantaloupe (Beaulieu and Grimm, 2001). Several sulfur volatiles have been detected in the aroma profiles of ripe melons and are thought to contribute to that fruit’s characteristic aroma (Wyllie and Leach, 1992). Due to this, some investigation into the biosynthesis of sulfur volatiles in general in melon fruits has been conducted. Isotope feeding experiments conducted with ^13^C_5_-L-methionine gave evidence that volatile *S*-methyl thioesters are formed through methionine catabolism (Gonda et al., 2013). This study found that the enzyme L-methionine-γ–lyase (MGL) was capable of cleaving radiolabeled methanethiol from L-methionine and that *S*-methyl thioesters incorporated a radiolabeled carbon at the methyl group. As it has been shown in strawberry that alcohol acyltransferase can catalyze the formation of straight-chain thioesters by condensing thioalcohols with acyl-CoAs (Noichinda et al., 1999b), it may be likely that branched-chain *S*-methyl thioesters in fruit arise from AAT-mediated condensation of branched-chain acyl-CoAs with methanethiol derived from the MGL-catalyzed cleavage of methionine (Figure 4). Empirically demonstrating if this process occurs in plants by incubating branched-chain volatile ester forming AAT enzymes with branched-chain acyl-CoAs and thioalcohols would answer an important question regarding the metabolism of volatile branched-chain sulfur compounds.

### What Biosynthetic Processes Are Capable of Forming Branched-Chain Alkanes in Fruit

Only one branched-chain alkane volatile was reported from all of the literature examined, the compound being 2-methylbutane and its presence being reported in raspberry (Aprea et al., 2015). Because the presence of this compound was reported below the level required for reliable quantification and because other studies of raspberry aroma failed to detect its presence, it is unlikely that that this compound plays a role in raspberry aroma. Nevertheless, as small to medium size branched alkanes in general are important components of commercial gasoline the biosynthesis of 2-methylbutane in fruit may be of interest to workers researching ways to bioengineer plants capable of producing fuel hydrocarbons. It has been shown that some plants can produce alkanes through the decarbonylation of aldehydes (Cheesbrough and Kolattukudy, 1984; Dennis and Kolattukudy, 1991; Aarts et al., 1995; Schneider-Belhaddad and Kolattukudy, 2000). While this route has been demonstrated to operate primarily on long- and very-long chain fatty acid derivatives in plants (Bernard et al., 2012; Ni et al., 2018), it may be a viable biochemical route to short branched-chain alkanes especially since branched-chain aldehydes are commonly found in several different kinds of plant (Supplementary Table 1). Further research with an emphasis on short branched-chain substrates is needed to confirm if such a pathway is indeed responsible for branched-chain alkane volatile biosynthesis in plants.

## Conclusion

While knowledge regarding the biochemical basis of branched-chain volatile metabolism in fruits has advanced significantly in recent years, it is still rather inadequate especially when compared to our detailed understanding of oxylipin, terpenoid, and phenylpropanoid volatile biosynthesis. Much more is known regarding the impact BCVs have on the flavor and aroma qualities of several edible fruits, yet this underlies the importance of obtaining a more thorough understanding of the molecular correlates underlying fruit BCV production. What has been published regarding this topic points to four general hypotheses concerning the mechanisms of BCV volatile biosynthesis in fruits, any or all of which might be operational *in vivo*. It is clear that properly elucidating which metabolic processes are responsible for fruit BCV biosynthesis will require a great deal of further experimental work, and it may very well be that the precise mechanisms vary from fruit to fruit.

Several questions regarding BCV metabolism remain unresolved, particularly those concerning the biosynthesis of more unusual compounds such as branched-chain alkanes and *S*-methyl thioesters. Furthermore, the regulation of important parts of several proposed BCV biosynthesis routes remains little known in plants. Whether well-studied regulatory mechanisms known to control similar pathways in mammals are also active in plants is a particularly relevant question, especially if bypassing these mechanisms presents a potential way to modulate BCV content in fruit tissue. Information gained on this aspect of BCV metabolism could prove quite useful to groups researching ways to improve the flavor of certain fruits by manipulating levels of important volatile compounds.

Ultimately, advancing our understanding of BCV metabolism represents a way to further our knowledge of the molecular basis of fruit flavor and aroma. The fact that important progress has been made should not detract from the fact that significant gaps remain regarding our understanding of how specifically these compounds are generated in fruits. Filling these gaps through rigorous experimental work will go a long way to making branched-chain volatiles as well understood as their oxylipin, terpenoid, and phenylpropanoid counterparts.

## Author Contributions

LB and PM conceived the idea to develop the manuscript. LB reviewed and wrote the original draft. DT and PM reviewed and helped to create the final version. All authors contributed to the article and approved the submitted version.

## Conflict of Interest

The authors declare that the research was conducted in the absence of any commercial or financial relationships that could be construed as a potential conflict of interest.

## Publisher’s Note

All claims expressed in this article are solely those of the authors and do not necessarily represent those of their affiliated organizations, or those of the publisher, the editors and the reviewers. Any product that may be evaluated in this article, or claim that may be made by its manufacturer, is not guaranteed or endorsed by the publisher.
