# Supplementary material for: Branched-Chain Volatiles in Fruit: A Molecular Perspective
Source: Front Plant Sci. 2022 Jan 27;12:814138. doi: 10.3389/fpls.2021.814138 (PMC8829073; doi:10.3389/fpls.2021.814138)
Supplement: Supplementary file 1 [file Table_1.DOCX]

**Supplementary Table 1.** List of branched-chain volatile compounds reported in 106 edible fruits across 175 published studies of fruit volatile content, listed by fruit. Compounds are identified by both name and CAS registry number. An absence of a CAS registry number for a compound is indicated by “N/A”. Reference indicates study where presence of compound was reported in the given fruit.

| **Name** | **CAS #** | **Reference** |
| --- | --- | --- |
| Abiu |  |  |
| 2-methyl-1-butanol | 137-32-6 | Faria et al., 2021 |
| 2-methylbutyl acetate | 624-41-9 | Faria et al., 2021 |
| 3-methyl-1-butanol | 123-51-3 | Faria et al., 2021 |
| 3-methylbutyl acetate | 123-92-2 | Faria et al., 2021 |
| Açaí |  |  |
| 3-methyl-1-butanol | 123-51-3 | Lim et al., 2016. |
| Acerola |  |  |
| 2-methylpropanal | 78-84-2 | Pino and Marbot, 2001 |
| 2-methylpropyl acetate | 110-19-0 | Vendramini and Trugo, 2000; Pino and Marbot, 2001 |
| 2-methylpropyl hexanoate | 105-79-3 | Nogueira et al., 2018 |
| 3-methyl-1-butanol | 123-51-3 | Pino and Marbot, 2001 |
| 3-methylbut-3-enyl 2-methylbut-2-enoate | 83783-87-3 | Pino and Marbot, 2001 |
| 3-methylbutanal | 590-86-3 | Pino and Marbot, 2001; Nogueira et al., 2018 |
| 3-methylbutyl 2-methylpropanoate | 2050-01-3 | Nogueira et al., 2018 |
| 3-methylbutyl acetate | 123-92-2 | Vendramini and Trugo, 2000; Nogueira et al., 2018 |
| 3-methylbutyl hexanoate | 2198-61-0 | Carasek and Pawliszyn, 2006; Nogueira et al., 2018 |
| ethyl 2-methylbut-2-enoate | 5837-78-5 | Nogueira et al., 2018 |
| ethyl 2-methylbutanoate | 7452-79-1 | Pino and Marbot, 2001; Nogueira et al., 2018 |
| ethyl 3-methylbutanoate | 108-64-5 | Pino and Marbot, 2001 |
| hexyl 2-methylbutanoate | 10032-15-2 | Nogueira et al., 2018 |
| hexyl 3-methylbutanoate | 10032-13-0 | Nogueira et al., 2018 |
| methyl 2-methylbutanoate | 868-57-5 | Pino and Marbot, 2001 |
| propyl 3-methylbutanoate | 557-00-6 | Nogueira et al., 2018 |
| Ackee |  |  |
| 3-methylbutanal | 590-86-3 | Grande-Tovar et al., 2019 |
| methyl 3-methylbutanoate | 556-24-1 | Grande-Tovar et al., 2019 |
| African star apple |  |  |
| 3-methyl-1-butanol | 123-51-3 | Lasekan et al., 2013 |
| Ambarella |  |  |
| (Z)-3-hexenyl 2-methylpropanoate | 41519-23-7 | Fraga and Rezende, 2001 |
| 2-methylpropyl 3-methylbutanoate | 589-59-3 | Fraga and Rezende, 2001 |
| 2-methylpropyl acetate | 110-19-0 | Fraga and Rezende, 2001 |
| 2-methylpropyl butanoate | 539-90-2 | Fraga and Rezende, 2001 |
| 3-methyl-1-butanol | 123-51-3 | Jayarathna et al., 2020 |
| ethyl 2-methylbut-2-enoate | 5837-78-5 | Fraga and Rezende, 2001 |
| ethyl 2-methylbutanoate | 7452-79-1 | Fraga and Rezende, 2001 |
| ethyl 2-methylpropanoate | 97-62-1 | Fraga and Rezende, 2001 |
| ethyl 3-methylbutanoate | 108-64-5 | Fraga and Rezende, 2001 |
| Apple |  |  |
| (E)-2-hexenyl 2-methylbutanoate | 94089-01-7 | Qin et al., 2017 |
| (Z)-3-hexenyl 2-methylbutanoate | 53398-85-9 | Qin et al., 2017 |
| 2-methyl-1-butanol | 137-32-6 | Dixon and Hewett, 2000; Qin et al., 2017; Liu et al., 2021 |
| 2-methylbutanoic acid | 116-53-0 | Liu et al., 2021 |
| 2-methylbutyl 2-methylbutanoate | 2445-78-5 | Qin et al., 2017; Liu et al., 2021 |
| 2-methylbutyl acetate | 624-41-9 | Dixon and Hewett, 2000; Qin et al., 2017; Liu et al., 2021 |
| 2-methylbutyl butanoate | 51115-64-1 | Qin et al., 2017; Liu et al., 2021 |
| 2-methylbutyl hexanoate | 2601-13-0 | Liu et al., 2021 |
| 2-methylbutyl octanoate | 67121-39-5 | Liu et al., 2021 |
| 2-methylbutyl propanoate | 2438-20-2 | Qin et al., 2017 |
| 2-methylpropyl acetate | 110-19-0 | Qin et al., 2017 |
| 3-methylbutyl 2-methylbutanoate | 27625-35-0 | Qin et al., 2017 |
| 3-methylbutyl hexanoate | 2198-61-0 | Qin et al., 2017 |
| butyl 2-methylbutanoate | 15706-73-7 | Dixon and Hewett, 2000; Qin et al., 2017; Liu et al., 2021 |
| ethyl 2-methylbutanoate | 7452-79-1 | Dixon and Hewett, 2000; Fraternale et al., 2011; Qin et al., 2017 |
| hexyl 2-methylbutanoate | 10032-15-2 | Dixon and Hewett, 2000; Qin et al., 2017; Liu et al., 2021 |
| hexyl 2-methylpropanoate | 2349-07-7 | Fraternale et al., 2011; Qin et al., 2017; Liu et al., 2021 |
| hexyl 3-methylbutanoate | 10032-13-0 | Fraternale et al., 2011 |
| methyl 2-methylbutanoate | 868-57-5 | Dixon and Hewett, 2000 |
| pentyl 2-methylbutanoate | 68039-26-9 | Qin et al., 2017; Liu et al., 2021 |
| pentyl 2-methylpropanoate | 2445-72-9 | Liu et al., 2021 |
| propyl 2-methylbutanoate | 37064-20-3 | Dixon and Hewett, 2000; Qin et al., 2017; Liu et al., 2021 |
| Apricot |  |  |
| (Z)-3-hexenyl 2-methylpropanoate | 41519-23-7 | Pintea et al., 2020 |
| (Z)-3-hexenyl 3-methylbutanoate | 35154-45-1 | Pintea et al., 2020 |
| 2-methylbutanoic acid | 116-53-0 | Xi et al., 2016 |
| 2-methylpropyl acetate | 110-19-0 | Pintea et al., 2020 |
| 3-methylbutanoic acid | 503-74-2 | Xi et al., 2016; Pintea et al., 2020 |
| 3-methylbutyl 2-methylpropanoate | 2050-01-3 | Pintea et al., 2020 |
| 3-methylbutyl acetate | 123-92-2 | Xi et al., 2016 |
| ethyl 2-methylbutanoate | 7452-79-1 | Pintea et al., 2020 |
| methyl 2-methylbutanoate | 868-57-5 | Pintea et al., 2020 |
| Araçá-boi |  |  |
| (Z)-3-hexenyl 3-methylbutanoate | 35154-45-1 | de Araújo et al., 2021 |
| 2-methylbutanoic acid | 116-53-0 | Pino and Quijano, 2007 |
| ethyl 2-methylbutanoate | 7452-79-1 | Pino and Quijano, 2007; de Araújo et al., 2021 |
| hexyl 2-methylbutanoate | 10032-15-2 | Pino and Quijano, 2007; de Araújo et al., 2021 |
| hexyl 2-methylpropanoate | 2349-07-7 | Pino and Quijano, 2007; de Araújo et al., 2021 |
| Australian brush cherry |  |  |
| 3-methylbutanal | 590-86-3 | Quijano-Célis et al., 2013 |
| hexyl 2-methylbutanoate | 10032-15-2 | Quijano-Célis et al., 2013 |
| Bacuri |  |  |
| 2-methyl-1-butanol | 137-32-6 | Boulanger et al., 1999 |
| 2-methyl-1-propanol | 78-83-1 | Uekane et al., 2017 |
| 3-methyl-1-butanol | 123-51-3 | Boulanger et al., 1999; Uekane et al., 2017 |
| 3-methylbutyl acetate | 123-92-2 | Uekane et al., 2017 |
| Banana |  |  |
| (E)-4-hepten-2-yl 3-methylbutanoate | N/A | Shiota, 1993 |
| (E)-4-octenyl 3-methylbutanoate | N/A | Shiota, 1993 |
| (Z)-4-decenyl 3-methylbutanoate | N/A | Shiota, 1993 |
| (Z)-4-hepten-2-yl 3-methylbutanoate | N/A | Shiota, 1993 |
| (Z)-4-octenyl 3-methylbutanoate | N/A | Shiota, 1993 |
| (Z)-5-octenyl 3-methylbutanoate | N/A | Shiota, 1993 |
| 2-methyl-1-propanol | 78-83-1 | Shiota, 1993; Nogueira et al., 2003 |
| 2-methylbutyl propanoate | 2438-20-2 | Zhu et al., 2018b |
| 2-methylpropyl 2-methylpropanoate | 97-85-8 | Zhu et al., 2018b |
| 2-methylpropyl 3-methylbutanoate | 589-59-3 | Shiota, 1993; Nogueira et al., 2003; Zhu et al., 2018b |
| 2-methylpropyl acetate | 110-19-0 | Shiota, 1993; Nogueira et al., 2003; Zhu et al., 2018b |
| 2-methylpropyl butanoate | 539-90-2 | Shiota, 1993; Nogueira et al., 2003; Zhu et al., 2018b |
| 2-methylpropyl hexanoate | 105-79-3 | Zhu et al., 2018b |
| 2-phenylethyl 3-methylbutanoate | 140-26-1 | Shiota, 1993 |
| 3-methyl-1-butanol | 123-51-3 | Shiota, 1993; Nogueira et al., 2003; Zhu et al., 2018b |
| 3-methylbutanal | 590-86-3 | Shiota, 1993 |
| 3-methylbutyl (E)-2-hexenoate | 72928-34-8 | Shiota, 1993 |
| 3-methylbutyl 2-methylbutanoate | 27625-35-0 | Shiota, 1993; Zhu et al., 2018b |
| 3-methylbutyl 2-methylpropanoate | 2050-01-3 | Shiota, 1993; Zhu et al., 2018b |
| 3-methylbutyl 3-methylbutanoate | 659-70-1 | Shiota, 1993; Nogueira et al., 2003; Zhu et al., 2018b |
| 3-methylbutyl acetate | 123-92-2 | Shiota, 1993; Nogueira et al., 2003; Zhu et al., 2018b |
| 3-methylbutyl butanoate | 106-27-4 | Shiota, 1993; Nogueira et al., 2003; Zhu et al., 2018b |
| 3-methylbutyl decanoate | 2306-91-4 | Zhu et al., 2018b |
| 3-methylbutyl hexanoate | 2198-61-0 | Shiota, 1993; Zhu et al., 2018b |
| 3-methylbutyl octanoate | 2035-99-6 | Zhu et al., 2018b |
| 3-methylbutyl pentanoate | 2050-09-1 | Shiota, 1993; Nogueira et al., 2003; Zhu et al., 2018b |
| 3-phenylpropyl 3-methylbutanoate | 5452-07-3 | Shiota, 1993 |
| butyl 3-methylbutanoate | 109-19-3 | Shiota, 1993 |
| hexan-2-yl 3-methylbut-2-enoate | N/A | Shiota, 1993 |
| hexyl 2-methylpropanoate | 2349-07-7 | Zhu et al., 2018b |
| hexyl 3-methylbutanoate | 10032-13-0 | Shiota, 1993; Zhu et al., 2018b |
| octyl 3-methylbutanoate | 7786-58-5 | Shiota, 1993 |
| pentan-2-yl 3-methylbut-2-enoate | 150462-84-3 | Shiota, 1993 |
| pentan-2-yl 3-methylbutanoate | 117421-34-8 | Shiota, 1993 |
| pentyl 3-methylbutanoate | 25415-62-7 | Zhu et al., 2018b |
| Bilberry |  |  |
| (Z)-3-hexenyl 3-methylbutanoate | 35154-45-1 | Rohloff et al., 2009 |
| 2-methylbutanal | 96-17-3 | Rohloff et al., 2009 |
| 3-methylbutyl acetate | 123-92-2 | Rohloff et al., 2009 |
| ethyl 2-hydroxy-3-methylbutanoate | 2441-06-7 | Rohloff et al., 2009 |
| ethyl 2-methylbutanoate | 7452-79-1 | Rohloff et al., 2009 |
| ethyl 2-methylpropanoate | 97-62-1 | Rohloff et al., 2009 |
| ethyl 3-hydroxy-3-methylbutanoate | 18267-36-2 | Rohloff et al., 2009 |
| ethyl 3-methylbutanoate | 108-64-5 | Rohloff et al., 2009 |
| hexyl 2-methylbutanoate | 10032-15-2 | Rohloff et al., 2009 |
| hexyl 3-methylbutanoate | 10032-13-0 | Rohloff et al., 2009 |
| methyl 2-hydroxy-3-methylbutanoate | 17417-00-4 | Rohloff et al., 2009 |
| methyl 3-methylbutanoate | 556-24-1 | Rohloff et al., 2009 |
| octyl 2-methylpropanoate | 109-15-9 | Rohloff et al., 2009 |
| Blackberry |  |  |
| 2-methyl-1-butanol | 137-32-6 | Qian and Wang, 2005 |
| 2-methyl-1-propanol | 78-83-1 | Qian and Wang, 2005 |
| 2-methylbutanal | 96-17-3 | Qian and Wang, 2005; D'Agostino et al., 2015 |
| 2-methylbutanoic acid | 116-53-0 | Qian and Wang, 2005; Du et al., 2010; D'Agostino et al., 2015 |
| 2-methylbutyl 3-methylbutanoate | 2445-77-4 | D'Agostino et al., 2015 |
| 3-methyl-1-butanol | 123-51-3 | Qian and Wang, 2005; D'Agostino et al., 2015 |
| 3-methylbutanal | 590-86-3 | Qian and Wang, 2005; D'Agostino et al., 2015 |
| 3-methylbutanoic acid | 503-74-2 | Qian and Wang, 2005; D'Agostino et al., 2015 |
| 3-methylbutyl acetate | 123-92-2 | Du et al., 2010 |
| ethyl 2-methylbutanoate | 7452-79-1 | Qian and Wang, 2005 |
| Blackcurrant |  |  |
| 2-(2-methylpropyl)-3-methoxypyrazine | 24683-00-9 | Jung, 2018 |
| 2-methyl-1-butanol | 137-32-6 | Jung, 2018 |
| 2-methyl-1-propanol | 78-83-1 | Jung, 2018 |
| 2-methylpropyl 3-methylbutanoate | 589-59-3 | Jung, 2018 |
| 3-methyl-1-butanol | 123-51-3 | Jung, 2018 |
| 3-methylbut-3-enyl 3-methylbutanoate | 54410-94-5 | Jung, 2018 |
| 3-methylbutyl 3-methylbutanoate | 659-70-1 | Jung, 2018 |
| butyl 2-methylbutanoate | 15706-73-7 | Harb et al., 2008 |
| ethyl 2-methylbutanoate | 7452-79-1 | Harb et al., 2008 |
| ethyl 3-methylbutanoate | 108-64-5 | Marsol-Vall et al., 2018 |
| hexyl 2-methylbutanoate | 10032-15-2 | Harb et al., 2008 |
| methyl 2-methylbutanoate | 868-57-5 | Marsol-Vall et al., 2018 |
| Blueberry |  |  |
| 2-methylbutanal | 96-17-3 | Farneti et al., 2017 |
| 2-methylbutyl acetate | 624-41-9 | Du et al., 2011 |
| 3-methyl-1-butanol | 123-51-3 | Gilbert et al., 2015; Farneti et al., 2017 |
| 3-methylbutanal | 590-86-3 | Gilbert et al., 2015; Farneti et al., 2017 |
| 3-methylbutyl acetate | 123-92-2 | Gilbert et al., 2015 |
| ethyl 2-hydroxy-3-methylbutanoate | 2441-06-7 | Farneti et al., 2017 |
| ethyl 2-methylbutanoate | 7452-79-1 | Du et al., 2011; Farneti et al., 2017; Cheng et al., 2020 |
| ethyl 2-methylpropanoate | 97-62-1 | Farneti et al., 2017 |
| ethyl 3-hydroxy-3-methylbutanoate | 18267-36-2 | Farneti et al., 2017 |
| ethyl 3-methylbutanoate | 108-64-5 | Du et al., 2011; Farneti et al., 2017 |
| methyl 2-methylbutanoate | 868-57-5 | Du et al., 2011; Farneti et al., 2017 |
| methyl 3-hydroxy-3-methylbutanoate | 6149-45-7 | Farneti et al., 2017 |
| methyl 3-methylbutanoate | 556-24-1 | Du et al., 2011; Gilbert et al., 2015; Farneti et al., 2017; Cheng et al., 2020 |
| Breadfruit |  |  |
| 2-methylbutanoic acid | 116-53-0 | Iwaoka et al., 1994 |
| 2-methylbutyl acetate | 624-41-9 | Faria et al., 2021 |
| 3-methylbutyl acetate | 123-92-2 | Faria et al., 2021 |
| Cajá |  |  |
| 2-methyl-1-propanol | 78-83-1 | Narain et al., 2004 |
| 2-methylbutyl acetate | 624-41-9 | Narain et al., 2004 |
| 2-methylpropanal | 78-84-2 | Narain et al., 2004 |
| 2-methylpropyl butanoate | 539-90-2 | Augusto et al., 2000; Ceva-Antunes et al., 2003 |
| 3-methylbutyl acetate | 123-92-2 | Augusto et al., 2000; Ceva-Antunes et al., 2003; Narain et al., 2004 |
| 3-methylbutyl butanoate | 106-27-4 | Augusto et al., 2000 |
| Calabura |  |  |
| 2-methyl-1-propanol | 78-83-1 | Wong et al., 1996a |
| 2-methylpropyl acetate | 110-19-0 | Wong et al., 1996a |
| 3-methyl-1-butanol | 123-51-3 | Wong et al., 1996a |
| 3-methylbutyl acetate | 123-92-2 | Wong et al., 1996a |
| ethyl 2-methylpropanoate | 97-62-1 | Wong et al., 1996a |
| Camu-camu |  |  |
| (Z)-3-hexenyl 2-methylbutanoate | 53398-85-9 | Quijano and Pino, 2007 |
| (Z)-3-hexenyl 2-methylpropanoate | 41519-23-7 | Quijano and Pino, 2007 |
| (Z)-3-hexenyl 3-methylbutanoate | 35154-45-1 | Quijano and Pino, 2007 |
| 2-phenylethyl 2-methylpropanoate | 103-48-0 | Quijano and Pino, 2007 |
| 3-methylbutanoic acid | 503-74-2 | Quijano and Pino, 2007 |
| 3-methylbutyl 3-methylbutanoate | 659-70-1 | Quijano and Pino, 2007 |
| 3-methylbutyl acetate | 123-92-2 | Quijano and Pino, 2007 |
| 3-methylbutyl hexanoate | 2198-61-0 | Quijano and Pino, 2007 |
| benzyl 3-methylbutanoate | 103-38-8 | Quijano and Pino, 2007 |
| ethyl 2-methylbutanoate | 7452-79-1 | Quijano and Pino, 2007 |
| ethyl 2-methylpropanoate | 97-62-1 | Quijano and Pino, 2007 |
| hexyl 2-methylbutanoate | 10032-15-2 | Quijano and Pino, 2007 |
| methyl 2-methylbutanoate | 868-57-5 | Quijano and Pino, 2007 |
| methyl 3-methylbutanoate | 556-24-1 | Quijano and Pino, 2007 |
| pentyl 2-methylpropanoate | 2445-72-9 | Quijano and Pino, 2007 |
| Canistel |  |  |
| 2-methylbutanoic acid | 116-53-0 | Pino, 2010 |
| 2-methylpropyl 2-hydroxypropanoate | 585-24-0 | Pino, 2010 |
| 3-methylbutyl acetate | 123-92-2 | Pino, 2010 |
| ethyl 2-methylbutanoate | 7452-79-1 | Pino, 2010 |
| ethyl 2-methylpropanoate | 97-62-1 | Pino, 2010 |
| methyl 2-methylbutanoate | 868-57-5 | Pino, 2010 |
| Cantaloupe |  |  |
| 2-methyl-1-butanol | 137-32-6 | Beaulieu and Grimm, 2001; Aubert and Bourger, 2004 |
| 2-methyl-1-propanol | 78-83-1 | Aubert and Bourger, 2004 |
| 2-methylbutyl 2-methylpropanoate | 2445-69-4 | Beaulieu and Grimm, 2001 |
| 2-methylbutyl 3-methylbutanoate | 2445-77-4 | Beaulieu and Grimm, 2001 |
| 2-methylbutyl acetate | 624-41-9 | Aubert and Bourger, 2004 |
| 2-methylbutyl butanoate | 51115-64-1 | Beaulieu and Grimm, 2001 |
| 2-methylpropyl acetate | 110-19-0 | Beaulieu and Grimm, 2001; Aubert and Bourger, 2004 |
| 2-methylpropyl butanoate | 539-90-2 | Beaulieu and Grimm, 2001 |
| 2-methylpropyl propanoate | 540-42-1 | Beaulieu and Grimm, 2001 |
| 3-methyl-1-butanol | 123-51-3 | Beaulieu and Grimm, 2001 |
| 3-methylbutyl acetate | 123-92-2 | Beaulieu and Grimm, 2001 |
| 3-methylbutyl butanoate | 106-27-4 | Beaulieu and Grimm, 2001 |
| 3-methylbutyl hexanoate | 2198-61-0 | Beaulieu and Grimm, 2001 |
| 3-methylbutyl octanoate | 2035-99-6 | Beaulieu and Grimm, 2001 |
| ethyl 2-methylbutanoate | 7452-79-1 | Beaulieu and Grimm, 2001; Aubert and Bourger, 2004 |
| ethyl 2-methylpropanoate | 97-62-1 | Beaulieu and Grimm, 2001 |
| methyl 2-methylbutanoate | 868-57-5 | Beaulieu and Grimm, 2001 |
| methyl 2-methylpropanoate | 547-63-7 | Beaulieu and Grimm, 2001 |
| propyl 2-methylbutanoate | 37064-20-3 | Beaulieu and Grimm, 2001 |
| *S*-methyl 3-methylbutanethioate | 23747-45-7 | Beaulieu and Grimm, 2001 |
| Cape gooseberry |  |  |
| 2-methyl-1-butanol | 137-32-6 | Yilmaztekin, 2014; Ramadan et al., 2015 |
| 2-methyl-1-propanol | 78-83-1 | Yilmaztekin, 2014 |
| 2-methylbutanal | 96-17-3 | Yilmaztekin, 2014 |
| 2-methylbutyl acetate | 624-41-9 | Yilmaztekin, 2014 |
| 2-methylpropanal | 78-84-2 | Majcher et al., 2020 |
| 2-methylpropyl butanoate | 539-90-2 | Yilmaztekin, 2014 |
| 2-methylpropyl octanoate | 5461-06-3 | Yilmaztekin, 2014 |
| 3-methylbutyl acetate | 123-92-2 | Yilmaztekin, 2014 |
| 3-methylbutyl decanoate | 2306-91-4 | Yilmaztekin, 2014 |
| 3-methylbutyl dodecanoate | 6309-51-9 | Yilmaztekin, 2014 |
| 3-methylbutyl octanoate | 2035-99-6 | Yilmaztekin, 2014 |
| ethyl 2-methylpropanoate | 97-62-1 | Majcher et al., 2020 |
| Cempedak |  |  |
| 2-(2-methylpropyl)-3-methoxypyrazine | 24683-00-9 | Grimm and Steinhaus, 2020 |
| 2-methyl-1-butanol | 137-32-6 | Grimm and Steinhaus, 2020 |
| 2-methylbutanal | 96-17-3 | Grimm and Steinhaus, 2020 |
| 2-methylpropanal | 78-84-2 | Grimm and Steinhaus, 2020 |
| 3-methyl-1-butanol | 123-51-3 | Grimm and Steinhaus, 2020 |
| 3-methylbutanal | 590-86-3 | Grimm and Steinhaus, 2020 |
| 3-methylbutanoic acid | 503-74-2 | Grimm and Steinhaus, 2020 |
| ethyl 2-methylbutanoate | 7452-79-1 | Grimm and Steinhaus, 2020 |
| ethyl 2-methylpropanoate | 97-62-1 | Grimm and Steinhaus, 2020 |
| ethyl 3-methylbutanoate | 108-64-5 | Grimm and Steinhaus, 2020 |
| methyl 3-methylbutanoate | 556-24-1 | Grimm and Steinhaus, 2020 |
| Champa |  |  |
| 2-methyl-1-propanol | 78-83-1 | Osorio et al., 2006 |
| 2-methylpropanoic acid | 79-31-2 | Osorio et al., 2006 |
| 3-methyl-1-butanol | 123-51-3 | Osorio et al., 2006 |
| 3-methylbutanoic acid | 503-74-2 | Osorio et al., 2006 |
| 3-methylbutyl acetate | 123-92-2 | Osorio et al., 2006 |
| methyl 2-hydroxy-3-methylbutanoate | 17417-00-4 | Osorio et al., 2006 |
| Cherimoya |  |  |
| 2-methyl-1-propanol | 78-83-1 | Pino and Roncal, 2016 |
| 3-methyl-1-butanol | 123-51-3 | Ferreira et al., 2009; Pino and Roncal, 2016 |
| 3-methylbutanal | 590-86-3 | Pino and Roncal, 2016 |
| 3-methylbutanoic acid | 503-74-2 | Ferreira et al., 2009 |
| 3-methylbutyl 3-methylbutanoate | 659-70-1 | Ferreira et al., 2009; Pino and Roncal, 2016 |
| 3-methylbutyl acetate | 123-92-2 | Ferreira et al., 2009 |
| 3-methylbutyl butanoate | 106-27-4 | Ferreira et al., 2009; Pino and Roncal, 2016 |
| butyl 3-methylbutanoate | 109-19-3 | Pino and Roncal, 2016 |
| ethyl 3-methylbutanoate | 108-64-5 | Pino and Roncal, 2016 |
| hexyl 2-methylpropanoate | 2349-07-7 | Ferreira et al., 2009; Pino and Roncal, 2016 |
| hexyl 3-methylbutanoate | 10032-13-0 | Ferreira et al., 2009; Pino and Roncal, 2016 |
| methyl 2-methylbutanoate | 868-57-5 | Pino and Roncal, 2016 |
| methyl 3-hydroxy-3-methylbutanoate | 6149-45-7 | Ferreira et al., 2009 |
| Cherry |  |  |
| 2-methylbutanal | 96-17-3 | Hayaloglu and Demir, 2016 |
| 2-methylbutanoic acid | 116-53-0 | Legua et al., 2017 |
| 2-methylpropanal | 78-84-2 | Sun et al., 2010 |
| 2-methylpropyl butanoate | 539-90-2 | Hayaloglu and Demir, 2016; Legua et al., 2017 |
| 2-methylpropyl hexanoate | 105-79-3 | Hayaloglu and Demir, 2016 |
| 3-methyl-1-butanol | 123-51-3 | Sun et al., 2010; Hayaloglu and Demir, 2016 |
| 3-methylbutanal | 590-86-3 | Sun et al., 2010; Hayaloglu and Demir, 2016 |
| 3-methylbutanoic acid | 503-74-2 | Sun et al., 2010; Hayaloglu and Demir, 2016 |
| 3-methylbutyl acetate | 123-92-2 | Hayaloglu and Demir, 2016 |
| 3-methylbutyl butanoate | 106-27-4 | Legua et al., 2017 |
| butyl 2-methylpropanoate | 97-87-0 | Hayaloglu and Demir, 2016 |
| ethyl 3-methylbutanoate | 108-64-5 | Hayaloglu and Demir, 2016 |
| Chinese bayberry |  |  |
| 2-methyl-1-butanol | 137-32-6 | Cheng et al., 2015 |
| 2-methylbutanoic acid | 116-53-0 | Cheng et al., 2015 |
| 2-methylpropanoic acid | 79-31-2 | Cheng et al., 2015 |
| 2-methylpropyl acetate | 110-19-0 | Cheng et al., 2015 |
| 3-methyl-1-butanol | 123-51-3 | Cheng et al., 2015; Fang et al., 2020 |
| 3-methylbutanoic acid | 503-74-2 | Kang et al., 2012; Cheng et al., 2015 |
| 3-methylbutyl acetate | 123-92-2 | Cheng et al., 2015; Fang et al., 2020 |
| ethyl 2-methylbut-2-enoate | 5837-78-5 | Cheng et al., 2015 |
| ethyl 2-methylpropanoate | 97-62-1 | Cheng et al., 2015 |
| ethyl 3-methylbut-2-enoate | 638-10-8 | Cheng et al., 2015 |
| Chinese quince |  |  |
| (Z)-3-hexenyl 2-methylbutanoate | 53398-85-9 | Choi et al., 2018 |
| 2-methylbutyl octanoate | 67121-39-5 | Choi et al., 2018 |
| 2-methylpropyl 2-methylbut-2-enoate | 7779-81-9 | Choi et al., 2018 |
| 2-methylpropyl 2-methylbutanoate | 2445-67-2 | Choi et al., 2018 |
| 2-methylpropyl 2-methylpropanoate | 97-85-8 | Choi et al., 2018 |
| 2-methylpropyl butanoate | 539-90-2 | Choi et al., 2018 |
| 2-methylpropyl decanoate | 30673-38-2 | Choi et al., 2018 |
| 2-methylpropyl octanoate | 5461-06-3 | Choi et al., 2018 |
| 3-methylbutyl hexanoate | 2198-61-0 | Choi et al., 2018 |
| 3-methylbutyl octanoate | 2035-99-6 | Choi et al., 2018 |
| butyl 2-methylbut-2-enoate | 7785-66-2 | Choi et al., 2018 |
| butyl 2-methylbutanoate | 15706-73-7 | Choi et al., 2018 |
| butyl 2-methylpropanoate | 97-87-0 | Choi et al., 2018 |
| ethyl 2-methylbutanoate | 7452-79-1 | Choi et al., 2018 |
| ethyl 2-methylpropanoate | 97-62-1 | Choi et al., 2018 |
| hexyl 2-methylbutanoate | 10032-15-2 | Choi et al., 2018 |
| octyl 2-methylpropanoate | 109-15-9 | Choi et al., 2018 |
| propyl 2-methylbutanoate | 37064-20-3 | Choi et al., 2018 |
| Chokeberry |  |  |
| 2-methylbutanal | 96-17-3 | Kraujalytė et al., 2013 |
| 3-methyl-1-butanol | 123-51-3 | Kraujalytė et al., 2013 |
| 3-methylbutanal | 590-86-3 | Kraujalytė et al., 2013 |
| ethyl 2-methylbutanoate | 7452-79-1 | Kraujalytė et al., 2013 |
| ethyl 3-methylbutanoate | 108-64-5 | Kraujalytė et al., 2013 |
| Cocona |  |  |
| 3-methyl-1-butanol | 123-51-3 | Faria et al., 2021 |
| 3-methylbutanal | 590-86-3 | Quijano and Pino, 2006 |
| ethyl 2-methylbutanoate | 7452-79-1 | Quijano and Pino, 2006 |
| hexyl 3-methylbutanoate | 10032-13-0 | Quijano and Pino, 2006 |
| methyl 3-methylbutanoate | 556-24-1 | Quijano and Pino, 2006 |
| Cranberry |  |  |
| 2-methyl-1-propanol | 78-83-1 | Zhu et al., 2016 |
| 2-methylbutanal | 96-17-3 | Zhu et al., 2016 |
| 2-methylbutanoic acid | 116-53-0 | Zhu et al., 2016 |
| 3-methyl-1-butanol | 123-51-3 | Ruse et al., 2012; Zhu et al., 2016 |
| 3-methylbutanal | 590-86-3 | Zhu et al., 2016 |
| 3-methylbutyl acetate | 123-92-2 | Zhu et al., 2016 |
| ethyl 2-methylbutanoate | 7452-79-1 | Zhu et al., 2016 |
| Cupuaçu |  |  |
| 1-methylethyl 2-methylpropanoate | 617-50-5 | Boulanger and Crouzet, 2000 |
| 2-methylpropyl 2-methylbutanoate | 2445-67-2 | Boulanger and Crouzet, 2000 |
| 2-methylpropyl 2-methylpropanoate | 97-85-8 | Boulanger and Crouzet, 2000 |
| 3-methyl-1-butanol | 123-51-3 | Boulanger and Crouzet, 2000 |
| 3-methylbutyl butanoate | 106-27-4 | Boulanger and Crouzet, 2000 |
| butyl 2-methylbutanoate | 15706-73-7 | Boulanger and Crouzet, 2000; Franco and Shibamoto, 2000 |
| butyl 3-methylbutanoate | 109-19-3 | Franco and Shibamoto, 2000 |
| ethyl 2-hydroxy-2-methylbutanoate | 77-70-3 | Boulanger and Crouzet, 2000 |
| ethyl 2-methylbutanoate | 7452-79-1 | Boulanger and Crouzet, 2000; Franco and Shibamoto, 2000 |
| Curuba |  |  |
| 2-methylpropyl acetate | 110-19-0 | Conde-Martínez et al., 2014 |
| Date |  |  |
| 3-methyl-1-butanol | 123-51-3 | Amira et al., 2011 |
| 3-methylbutyl acetate | 123-92-2 | Amira et al., 2011 |
| hexyl 3-methylbutanoate | 10032-13-0 | Amira et al., 2011 |
| Duku |  |  |
| 2-methyl-1-propanol | 78-83-1 | Wong et al., 1994 |
| 3-methyl-1-butanol | 123-51-3 | Wong et al., 1994 |
| Durian |  |  |
| 2-(2-methylpropyl)-3-methoxypyrazine | 24683-00-9 | Li et al., 2012 |
| 2-methyl-1-butanol | 137-32-6 | Belgis et al., 2017 |
| 2-methylbutanoic acid | 116-53-0 | Irwandi et al., 2008; Li et al., 2012; Belgis et al., 2017 |
| 3-methyl-1-butanol | 123-51-3 | Belgis et al., 2017 |
| ethyl 2-hydroxy-2-methylbutanoate | 77-70-3 | Li et al., 2012 |
| ethyl 2-methylbutanoate | 7452-79-1 | Irwandi et al., 2008; Li et al., 2012; Belgis et al., 2017 |
| ethyl 2-methylpropanoate | 97-62-1 | Li et al., 2012 |
| methyl 2-methylbutanoate | 868-57-5 | Li et al., 2012; Belgis et al., 2017 |
| propyl 2-methylbutanoate | 37064-20-3 | Li et al., 2012; Belgis et al., 2017 |
| propyl 3-methylbutanoate | 557-00-6 | Belgis et al., 2017 |
| Elderberry |  |  |
| 3-methyl-1-butanol | 123-51-3 | Ağalar et al., 2014 |
| 3-methylbutyl acetate | 123-92-2 | Ağalar et al., 2014 |
| Fig |  |  |
| 2-methyl-1-propanol | 78-83-1 | Russo et al., 2017 |
| 2-methylbutanal | 96-17-3 | Russo et al., 2017 |
| 2-methylpropanal | 78-84-2 | Russo et al., 2017 |
| 2-methylpropyl acetate | 110-19-0 | Russo et al., 2017 |
| 3-methyl-1-butanol | 123-51-3 | Russo et al., 2017 |
| 3-methylbutanal | 590-86-3 | Russo et al., 2017 |
| 3-methylbutyl acetate | 123-92-2 | Russo et al., 2017 |
| ethyl 2-methylbutanoate | 7452-79-1 | Russo et al., 2017 |
| ethyl 3-methylbutanoate | 108-64-5 | Russo et al., 2017 |
| Gabiroba |  |  |
| 2-methyl-1-propanol | 78-83-1 | Ferreira et al., 2016 |
| 3-methyl-1-butanol | 123-51-3 | Ferreira et al., 2016 |
| ethyl 2-methylbutanoate | 7452-79-1 | Ferreira et al., 2016 |
| ethyl 3-methylbutanoate | 108-64-5 | Ferreira et al., 2016 |
| Genipap |  |  |
| 2-methyl-1-butanol | 137-32-6 | Pinto et al., 2006 |
| 2-methylbutanoic acid | 116-53-0 | Borges and Rezende, 2000; Pinto et al., 2006 |
| 2-methylpropanoic acid | 79-31-2 | Pinto et al., 2006 |
| 3-methyl-1-butanol | 123-51-3 | Pinto et al., 2006 |
| 3-methylbutanoic acid | 503-74-2 | Borges and Rezende, 2000; Pinto et al., 2006 |
| ethyl 2-methylbut-2-enoate | 5837-78-5 | Borges and Rezende, 2000 |
| ethyl 2-methylbutanoate | 7452-79-1 | Borges and Rezende, 2000 |
| ethyl 3-methylbutanoate | 108-64-5 | Borges and Rezende, 2000 |
| methyl 2-methylbut-2-enoate | 6622-76-0 | Borges and Rezende, 2000 |
| methyl 2-methylbutanoate | 868-57-5 | Pinto et al., 2006 |
| methyl 2-methylpropanoate | 547-63-7 | Borges and Rezende, 2000 |
| methyl 3-methylbutanoate | 556-24-1 | Pinto et al., 2006 |
| Goji berry |  |  |
| 2-methylbutyl acetate | 624-41-9 | Lu et al., 2017 |
| 3-methyl-1-butanol | 123-51-3 | Lu et al., 2017 |
| hexyl 2-methylbutanoate | 10032-15-2 | Lu et al., 2017 |
| Grape |  |  |
| 2-methylbutanal | 96-17-3 | Wu et al., 2016 |
| 3-methylbutanal | 590-86-3 | Wu et al., 2016 |
| ethyl 2-methylbut-2-enoate | 5837-78-5 | Yang et al., 2011 |
| ethyl 2-methylbutanoate | 7452-79-1 | Yang et al., 2011 |
| ethyl 2-methylpropanoate | 97-62-1 | Wu et al., 2016 |
| ethyl 3-methylbutanoate | 108-64-5 | Wu et al., 2016 |
| Grosella |  |  |
| benzyl 3-methylbutanoate | 103-38-8 | Pino et al., 2008 |
| hexyl 2-methylpropanoate | 2349-07-7 | Pino et al., 2008 |
| Guava |  |  |
| (Z)-3-hexenyl 2-methylpropanoate | 41519-23-7 | Elizalde-González and Segura-Rivera, 2018 |
| 2-methyl-1-butanol | 137-32-6 | Pino and Bent, 2013 |
| 2-methyl-1-propanol | 78-83-1 | Pino and Bent, 2013 |
| 2-methylbutanoic acid | 116-53-0 | Pino and Bent, 2013 |
| 2-methylpropanoic acid | 79-31-2 | Pino and Bent, 2013 |
| 2-methylpropyl acetate | 110-19-0 | Pino and Bent, 2013 |
| 3-methyl-1-butanol | 123-51-3 | Pino and Bent, 2013 |
| 3-methylbutanal | 590-86-3 | Pino and Bent, 2013 |
| 3-methylbutyl acetate | 123-92-2 | Elizalde-González and Segura-Rivera, 2018 |
| 3-phenylpropyl 2-methylpropanoate | 103-58-2 | Pino and Bent, 2013 |
| butyl 2-methylbutanoate | 15706-73-7 | Elizalde-González and Segura-Rivera, 2018 |
| Jabuticaba |  |  |
| 2-methylpropyl acetate | 110-19-0 | Sanabria et al., 2018 |
| 3-methyl-1-butanol | 123-51-3 | Plagemann et al., 2012; Sanabria et al., 2018 |
| 3-methylbutanal | 590-86-3 | Sanabria et al., 2018 |
| 3-methylbutyl acetate | 123-92-2 | Sanabria et al., 2018; Freitas et al., 2020 |
| ethyl 2-methylbut-2-enoate | 5837-78-5 | Freitas et al., 2020 |
| ethyl 2-methylbutanoate | 7452-79-1 | Freitas et al., 2020 |
| ethyl 3-methylbutanoate | 108-64-5 | Freitas et al., 2020 |
| propyl 2-methylprop-2-enoate | 2210-28-8 | Freitas et al., 2020 |
| Jackfruit |  |  |
| 2-(2-methylpropyl)-3-methoxypyrazine | 24683-00-9 | Grimm and Steinhaus, 2019 |
| 2-methyl-1-butanol | 137-32-6 | Ong et al., 2008; Grimm and Steinhaus, 2019 |
| 2-methyl-1-propanol | 78-83-1 | Ong et al., 2008 |
| 2-methylbutanal | 96-17-3 | Ong et al., 2008; Grimm and Steinhaus, 2019 |
| 2-methylbutyl acetate | 624-41-9 | Maia et al., 2004; Ong et al., 2008 |
| 2-methylpropanal | 78-84-2 | Grimm and Steinhaus, 2019 |
| 2-methylpropyl 3-methylbutanoate | 589-59-3 | Maia et al., 2004; Ong et al., 2008 |
| 2-methylpropyl acetate | 110-19-0 | Ong et al., 2008 |
| 2-phenylethyl 3-methylbutanoate | 140-26-1 | Maia et al., 2004 |
| 3-methyl-1-butanol | 123-51-3 | Maia et al., 2004; Ong et al., 2008; Grimm and Steinhaus, 2019 |
| 3-methylbutanal | 590-86-3 | Ong et al., 2008; Grimm and Steinhaus, 2019 |
| 3-methylbutanoic acid | 503-74-2 | Grimm and Steinhaus, 2019 |
| 3-methylbutyl 3-methylbutanoate | 659-70-1 | Maia et al., 2004; Ong et al., 2008 |
| 3-methylbutyl acetate | 123-92-2 | Maia et al., 2004; Ong et al., 2008; Grimm and Steinhaus, 2019 |
| 3-methylbutyl butanoate | 106-27-4 | Maia et al., 2004; Ong et al., 2008 |
| 3-methylbutyl hexanoate | 2198-61-0 | Maia et al., 2004 |
| butyl 3-methylbutanoate | 109-19-3 | Maia et al., 2004; Ong et al., 2008 |
| ethyl 2-methylbutanoate | 7452-79-1 | Maia et al., 2004; Ong et al., 2008; Grimm and Steinhaus, 2019 |
| ethyl 2-methylpropanoate | 97-62-1 | Grimm and Steinhaus, 2019 |
| ethyl 3-methylbutanoate | 108-64-5 | Maia et al., 2004; Ong et al., 2008; Grimm and Steinhaus, 2019 |
| hexyl 3-methylbutanoate | 10032-13-0 | Maia et al., 2004 |
| methyl 3-methylbutanoate | 556-24-1 | Maia et al., 2004; Ong et al., 2008; Grimm and Steinhaus, 2019 |
| pentyl 3-methylbutanoate | 25415-62-7 | Ong et al., 2008 |
| propyl 3-methylbutanoate | 557-00-6 | Maia et al., 2004; Ong et al., 2008 |
| Jujube |  |  |
| 2-methyl-1-butanol | 137-32-6 | Yang et al., 2019 |
| 2-methyl-1-propanol | 78-83-1 | Yang et al., 2019 |
| 2-methylbutanoic acid | 116-53-0 | Yang et al., 2019 |
| 2-methylpropanoic acid | 79-31-2 | Chen et al., 2018; Yang et al., 2019 |
| 3-methyl-1-butanol | 123-51-3 | Yang et al., 2019 |
| 3-methylbutanoic acid | 503-74-2 | Chen et al., 2018 |
| ethyl 2-methylpropanoate | 97-62-1 | Yang et al., 2019 |
| Kiwifruit |  |  |
| 2-methylbutanal | 96-17-3 | Zhang et al., 2016 |
| 2-methylbutanoic acid | 116-53-0 | Lindhorst and Steinhaus, 2016 |
| 2-methylpropanal | 78-84-2 | Zhang et al., 2016 |
| 2-methylpropyl acetate | 110-19-0 | Lindhorst and Steinhaus, 2016 |
| 2-methylpropyl butanoate | 539-90-2 | Zhang et al., 2019a |
| 3-methylbutanal | 590-86-3 | Zhang et al., 2016 |
| 3-methylbutanoic acid | 503-74-2 | Lindhorst and Steinhaus, 2016 |
| ethyl 2-methylbutanoate | 7452-79-1 | Lindhorst and Steinhaus, 2016 |
| ethyl 2-methylpropanoate | 97-62-1 | Cozzolino et al., 2020 |
| methyl 2-methylbutanoate | 868-57-5 | Cozzolino et al., 2020 |
| methyl 2-methylpropanoate | 547-63-7 | Cozzolino et al., 2020; Zhao et al., 2021 |
| Kukumakranka |  |  |
| 1-methylethyl 2-methylpropanoate | 617-50-5 | Kamatou et al., 2008 |
| 2-methylbutyl 3-methylbutanoate | 2445-77-4 | Kamatou et al., 2008 |
| 2-methylbutyl benzoate | 52513-03-8 | Kamatou et al., 2008 |
| 2-methylbutyl butanoate | 51115-64-1 | Kamatou et al., 2008 |
| 2-methylpropyl 2-methylpropanoate | 97-85-8 | Kamatou et al., 2008 |
| 2-methylpropyl 3-methylbutanoate | 589-59-3 | Kamatou et al., 2008 |
| 2-methylpropyl benzoate | 120-50-3 | Kamatou et al., 2008 |
| 2-methylpropyl butanoate | 539-90-2 | Kamatou et al., 2008 |
| 2-methylpropyl hexanoate | 105-79-3 | Kamatou et al., 2008 |
| 3-methylbutyl acetate | 123-92-2 | Kamatou et al., 2008 |
| 3-methylbutyl benzoate | 94-46-2 | Kamatou et al., 2008 |
| 3-methylbutyl hexanoate | 2198-61-0 | Kamatou et al., 2008 |
| 3-methylbutyl octanoate | 2035-99-6 | Kamatou et al., 2008 |
| benzyl 3-methylbutanoate | 103-38-8 | Kamatou et al., 2008 |
| ethyl 3-methylbutanoate | 108-64-5 | Kamatou et al., 2008 |
| heptyl 2-methylpropanoate | 2349-13-5 | Kamatou et al., 2008 |
| hexyl 3-methylbutanoate | 10032-13-0 | Kamatou et al., 2008 |
| neryl 2-methylpropanoate | 2345-24-6 | Kamatou et al., 2008 |
| octyl 3-methylbutanoate | 7786-58-5 | Kamatou et al., 2008 |
| Kundang |  |  |
| 2-methyl-1-butanol | 137-32-6 | Wong et al., 1996b |
| 2-methyl-1-propanol | 78-83-1 | Wong et al., 1996b |
| 3-methyl-1-butanol | 123-51-3 | Wong et al., 1996b |
| Langsat |  |  |
| 2-methyl-1-propanol | 78-83-1 | Wong et al., 1994 |
| 3-methylbutyl acetate | 123-92-2 | Wong et al., 1994 |
| ethyl 2-methylbutanoate | 7452-79-1 | Wong et al., 1994 |
| ethyl 2-methylpropanoate | 97-62-1 | Wong et al., 1994 |
| methyl 2-methylbutanoate | 868-57-5 | Wong et al., 1994 |
| Lingonberry |  |  |
| 2-methyl-1-butanol | 137-32-6 | Viljanen et al., 2014 |
| 2-methylbutanoic acid | 116-53-0 | Viljanen et al., 2014 |
| 3-methyl-1-butanol | 123-51-3 | Viljanen et al., 2014 |
| 3-methylbutanoic acid | 503-74-2 | Viljanen et al., 2014 |
| 3-methylbutyl acetate | 123-92-2 | Viljanen et al., 2014 |
| Longan |  |  |
| 2-methyl-1-propanol | 78-83-1 | Wong et al., 1996c |
| 3-methyl-1-butanol | 123-51-3 | Wong et al., 1996c |
| ethyl 2-methylbutanoate | 7452-79-1 | Wong et al., 1996c |
| ethyl 2-methylpropanoate | 97-62-1 | Wong et al., 1996c |
| ethyl 3-methylbutanoate | 108-64-5 | Wong et al., 1996c |
| methyl 2-hydroxy-3-methylbutanoate | 17417-00-4 | Wong et al., 1996c |
| methyl 3-hydroxy-3-methylbutanoate | 6149-45-7 | Wong et al., 1996c |
| Loquat |  |  |
| 2-methyl-1-butanol | 137-32-6 | Pino et al., 2002 |
| 2-methyl-1-propanol | 78-83-1 | Pino et al., 2002 |
| 2-methylbutanoic acid | 116-53-0 | Besada et al., 2017 |
| 2-phenoxyethyl 2-methylpropanoate | 103-60-6 | Besada et al., 2017 |
| 3-methyl-1-butanol | 123-51-3 | Pino et al., 2002 |
| ethyl 3-hydroxy-3-methylbutanoate | 18267-36-2 | Pino et al., 2002 |
| methyl 2-methylbutanoate | 868-57-5 | Besada et al., 2017 |
| Lulo |  |  |
| 2-methylpropanal | 78-84-2 | Corpas et al., 2016 |
| 2-methylpropyl acetate | 110-19-0 | Corpas et al., 2016 |
| 2-methylpropyl hexanoate | 105-79-3 | Corpas et al., 2016 |
| Lychee |  |  |
| 3-methyl-1-butanol | 123-51-3 | Wu et al., 2009 |
| 3-methylbutanoic acid | 503-74-2 | Ong and Acree, 1998; Feng et al., 2018 |
| 3-methylbutyl acetate | 123-92-2 | Ong and Acree, 1998; Wu et al., 2009 |
| ethyl 2-methylbutanoate | 7452-79-1 | Ong and Acree, 1998 |
| ethyl 2-methylpropanoate | 97-62-1 | Ong and Acree, 1998 |
| Mamey sapote |  |  |
| 2-methyl-1-propanol | 78-83-1 | Martín and Osorio, 2019 |
| 3-methylbutanal | 590-86-3 | Martín and Osorio, 2019 |
| 3-methylbutanoic acid | 503-74-2 | Martín and Osorio, 2019 |
| Mandarin |  |  |
| 2-methyl-1-butanol | 137-32-6 | Xiao et al., 2017 |
| 2-methylpropyl acetate | 110-19-0 | Xiao et al., 2017 |
| 3-methyl-1-butanol | 123-51-3 | Xiao et al., 2017 |
| 3-methylbutyl acetate | 123-92-2 | Xiao et al., 2017 |
| ethyl 2-methylbutanoate | 7452-79-1 | Tietel et al., 2011; Xiao et al., 2017 |
| ethyl 2-methylpropanoate | 97-62-1 | Xiao et al., 2017 |
| ethyl 3-methylbutanoate | 108-64-5 | Xiao et al., 2017 |
| Mangaba |  |  |
| 3-methyl-1-butanol | 123-51-3 | Sampaio and Nogueira, 2006 |
| 3-methylbutyl acetate | 123-92-2 | Sampaio and Nogueira, 2006 |
| ethyl 2-methylbutanoate | 7452-79-1 | Narain et al., 2010 |
| pentyl 2-methylpropanoate | 2445-72-9 | Narain et al., 2010 |
| Mango |  |  |
| (Z)-3-hexenyl 2-methylbutanoate | 53398-85-9 | Pino et al., 2005 |
| 2-methyl-1-butanol | 137-32-6 | Pino et al., 2005 |
| 2-methyl-1-propanol | 78-83-1 | Pino et al., 2005 |
| 2-methylbutanoic acid | 116-53-0 | Pino et al., 2005 |
| 2-methylbutyl acetate | 624-41-9 | Pino et al., 2005 |
| 2-methylpropanoic acid | 79-31-2 | Pino et al., 2005 |
| 2-methylpropyl 2-methylbut-2-enoate | 7779-81-9 | Pino et al., 2005 |
| 2-methylpropyl dodecanoate | 37811-72-6 | Pino et al., 2005 |
| 2-methylpropyl hexadecanoate | 110-34-9 | Pino et al., 2005 |
| 2-methylpropyl phenylacetate | 102-13-6 | Pino et al., 2005 |
| 2-phenylethyl 2-methylbut-2-enoate | 55719-85-2 | Pino et al., 2005 |
| 3-methyl-1-butanol | 123-51-3 | Pino et al., 2005 |
| 3-methylbutanoic acid | 503-74-2 | Pino et al., 2005 |
| 3-methylbutyl acetate | 123-92-2 | Pino et al., 2005 |
| 3-methylbutyl butanoate | 106-27-4 | Pino et al., 2005 |
| 3-methylbutyl hexadecanoate | 81974-61-0 | Pino et al., 2005 |
| 3-methylbutyl hexanoate | 2198-61-0 | Pino et al., 2005 |
| benzyl 3-methylbutanoate | 103-38-8 | Pino et al., 2005 |
| butyl 2-methylpropanoate | 97-87-0 | Pino et al., 2005 |
| butyl 3-methylbutanoate | 109-19-3 | Pino et al., 2005 |
| decyl 2-methylpropanoate | 5454-22-8 | Pino et al., 2005 |
| ethyl 2-methylbut-2-enoate | 5837-78-5 | Pino et al., 2005 |
| ethyl 2-methylbutanoate | 7452-79-1 | Pino et al., 2005 |
| ethyl 2-methylprop-2-enoate | 97-63-2 | Pino et al., 2005 |
| ethyl 2-methylpropanoate | 97-62-1 | Pino et al., 2005 |
| ethyl 3-methylbutanoate | 108-64-5 | Pino et al., 2005 |
| methyl 2-methylbutanoate | 868-57-5 | Pino et al., 2005 |
| methyl 2-methylprop-2-enoate | 80-62-6 | Pino et al., 2005 |
| octyl 2-methylpropanoate | 109-15-9 | Pino et al., 2005 |
| pentyl 2-methylpropanoate | 2445-72-9 | Pino et al., 2005 |
| Marula |  |  |
| 3-methylbutyl hexanoate | 2198-61-0 | Viljoen et al., 2008 |
| 3-methylbutyl octanoate | 2035-99-6 | Viljoen et al., 2008 |
| benzyl 2-methylbut-2-enoate | 37526-88-8 | Viljoen et al., 2008 |
| ethyl 3-methylbutanoate | 108-64-5 | Viljoen et al., 2008 |
| Mobola plum |  |  |
| 2-methylpropyl butanoate | 539-90-2 | Joulain et al., 2004 |
| 2-phenylethyl 2-methylbutanoate | 24817-51-4 | Joulain et al., 2004 |
| 2-phenylethyl 2-methylpropanoate | 103-48-0 | Joulain et al., 2004 |
| 2-phenylethyl 3-methylbutanoate | 140-26-1 | Joulain et al., 2004 |
| 3-methyl-1-butanol | 123-51-3 | Joulain et al., 2004 |
| 3-methylbutyl 2-aminobenzoate | 28457-05-8 | Joulain et al., 2004 |
| 3-methylbutyl acetate | 123-92-2 | Joulain et al., 2004 |
| 3-methylbutyl butanoate | 106-27-4 | Joulain et al., 2004 |
| 3-methylbutyl propanoate | 105-68-0 | Joulain et al., 2004 |
| butyl 2-methylbutanoate | 15706-73-7 | Joulain et al., 2004 |
| butyl 3-methylbutanoate | 109-19-3 | Joulain et al., 2004 |
| ethyl 2-methylbut-2-enoate | 5837-78-5 | Joulain et al., 2004 |
| ethyl 2-methylbutanoate | 7452-79-1 | Joulain et al., 2004 |
| methyl 3-methylbutanoate | 556-24-1 | Joulain et al., 2004 |
| propyl 2-methylbutanoate | 37064-20-3 | Joulain et al., 2004 |
| propyl 2-methylpropanoate | 644-49-5 | Joulain et al., 2004 |
| propyl 3-methylbutanoate | 557-00-6 | Joulain et al., 2004 |
| Monkey orange |  |  |
| 2-methylbutyl acetate | 624-41-9 | Shoko et al., 2013 |
| 2-methylpropyl acetate | 110-19-0 | Shoko et al., 2013 |
| Mulberry |  |  |
| 2-methylbutanal | 96-17-3 | Zhu et al., 2018a |
| 2-methylpropyl benzoate | 120-50-3 | Chen et al., 2015 |
| 3-methyl-1-butanol | 123-51-3 | Zhu et al., 2018a |
| 3-methylbutanal | 590-86-3 | Zhu et al., 2018a |
| ethyl 2-methylbutanoate | 7452-79-1 | Zhu et al., 2018a |
| Murici |  |  |
| 2-methyl-1-propanol | 78-83-1 | Alves and Franco, 2003; Rezende and Fraga, 2003; Uekane et al., 2017 |
| 2-methylpropanoic acid | 79-31-2 | Alves and Franco, 2003; Uekane et al., 2017 |
| 3-methyl-1-butanol | 123-51-3 | Alves and Franco, 2003; Rezende and Fraga, 2003; Uekane et al., 2017 |
| 3-methylbutanoic acid | 503-74-2 | Uekane et al., 2017 |
| 3-methylbutyl 2-methylpropanoate | 2050-01-3 | Alves and Franco, 2003 |
| 3-methylbutyl acetate | 123-92-2 | Alves and Franco, 2003; Uekane et al., 2017 |
| Murtilla |  |  |
| 3-methylbutyl acetate | 123-92-2 | Scheuermann et al., 2008 |
| ethyl 2-methylbutanoate | 7452-79-1 | Scheuermann et al., 2008 |
| ethyl 2-methylpropanoate | 97-62-1 | Scheuermann et al., 2008 |
| ethyl 3-methylbutanoate | 108-64-5 | Scheuermann et al., 2008 |
| methyl 2-methylbutanoate | 868-57-5 | Scheuermann et al., 2008 |
| Noni berry |  |  |
| 2-methyl-1-butanol | 137-32-6 | Pino et al., 2010 |
| 2-methylbutanoic acid | 116-53-0 | Pino et al., 2010 |
| 2-methylpropanoic acid | 79-31-2 | Pino et al., 2010 |
| 2-methylpropyl hexanoate | 105-79-3 | Pino et al., 2010 |
| 3-methyl-1-butanol | 123-51-3 | Pino et al., 2010 |
| 3-methylbut-3-enyl 2-methylpropanoate | 76649-23-5 | Pino et al., 2010; Wall et al., 2018 |
| 3-methylbut-3-enyl 3-methylbutanoate | 54410-94-5 | Pino et al., 2010; Wall et al., 2018 |
| 3-methylbutanoic acid | 503-74-2 | Pino et al., 2010 |
| 3-methylbutyl hexanoate | 2198-61-0 | Pino et al., 2010 |
| 3-methylbutyl octanoate | 2035-99-6 | Pino et al., 2010 |
| butyl 2-methylpropanoate | 97-87-0 | Pino et al., 2010 |
| ethyl 2-methylbutanoate | 7452-79-1 | Pino et al., 2010 |
| ethyl 2-methylpropanoate | 97-62-1 | Pino et al., 2010 |
| hexyl 2-methylpropanoate | 2349-07-7 | Pino et al., 2010 |
| Ocorocillo |  |  |
| (Z)-3-hexenyl 2-methylpropanoate | 41519-23-7 | da Silva et al., 2019a |
| Papaya |  |  |
| 2-methyl-1-butanol | 137-32-6 | Pino, 2014 |
| 2-methyl-1-propanol | 78-83-1 | Pino, 2014 |
| 2-methylpropyl acetate | 110-19-0 | da Rocha et al., 2017 |
| 3-methyl-1-butanol | 123-51-3 | Pino, 2014 |
| 3-methylbutanal | 590-86-3 | Pino, 2014 |
| 3-methylbutyl acetate | 123-92-2 | da Rocha et al., 2017 |
| 3-methylbutyl butanoate | 106-27-4 | Pino, 2014 |
| 3-methylbutyl hexanoate | 2198-61-0 | Pino, 2014 |
| ethyl 3-methylbutanoate | 108-64-5 | Pino, 2014 |
| methyl 2-hydroxy-2-methylbutanoate | 32793-34-3 | Pino, 2014 |
| methyl 2-hydroxy-3-methylbutanoate | 17417-00-4 | Pino, 2014 |
| methyl 3-methylbut-2-enoate | 924-50-5 | Pino, 2014 |
| Pariri |  |  |
| methyl 2-hydroxy-3-methylbutanoate | 17417-00-4 | Maia et al., 2003 |
| methyl 2-methylbutanoate | 868-57-5 | Maia et al., 2003 |
| Passionfruit |  |  |
| 2-methyl-1-butanol | 137-32-6 | Werkhoff et al., 1998 |
| 2-methyl-1-propanol | 78-83-1 | Werkhoff et al., 1998 |
| 2-methylbutanal | 96-17-3 | Werkhoff et al., 1998 |
| 2-methylbutanoic acid | 116-53-0 | Werkhoff et al., 1998 |
| 2-methylbutyl acetate | 624-41-9 | Werkhoff et al., 1998 |
| 2-methylbutyl butanoate | 51115-64-1 | Werkhoff et al., 1998 |
| 2-methylpropyl acetate | 110-19-0 | Werkhoff et al., 1998; Janzantti and Monteiro, 2017 |
| 2-methylpropyl butanoate | 539-90-2 | Werkhoff et al., 1998 |
| 2-methylpropyl hexanoate | 105-79-3 | Carasek and Pawliszyn, 2006 |
| 2-methylpropyl octanoate | 5461-06-3 | Li et al., 2021 |
| 3-methyl-1-butanol | 123-51-3 | Werkhoff et al., 1998 |
| 3-methylbutanal | 590-86-3 | Werkhoff et al., 1998 |
| 3-methylbutanoic acid | 503-74-2 | Werkhoff et al., 1998 |
| 3-methylbutyl acetate | 123-92-2 | Werkhoff et al., 1998 |
| 3-methylbutyl butanoate | 106-27-4 | Werkhoff et al., 1998 |
| 3-methylbutyl hexanoate | 2198-61-0 | Li et al., 2021 |
| ethyl 2-methylbutanoate | 7452-79-1 | Werkhoff et al., 1998 |
| hexyl 2-methylbutanoate | 10032-15-2 | Werkhoff et al., 1998 |
| hexyl 2-methylpropanoate | 2349-07-7 | Werkhoff et al., 1998 |
| hexyl 3-methylbutanoate | 10032-13-0 | Werkhoff et al., 1998 |
| methyl 2-methylbutanoate | 868-57-5 | Janzantti and Monteiro, 2017 |
| neryl 2-methylpropanoate | 2345-24-6 | Werkhoff et al., 1998 |
| Peach |  |  |
| 2-methylbutanoic acid | 116-53-0 | Sánchez et al., 2012 |
| 2-methylpropyl acetate | 110-19-0 | Sánchez et al., 2012 |
| 3-methylbutanoic acid | 503-74-2 | Sánchez et al., 2012 |
| Pear |  |  |
| 2-methyl-1-butanol | 137-32-6 | Qin et al., 2012; Wang et al., 2019 |
| 2-methyl-1-propanol | 78-83-1 | Wang et al., 2019 |
| 2-methylbutyl 2-methylbutanoate | 2445-78-5 | Wang et al., 2019 |
| 2-methylbutyl acetate | 624-41-9 | Qin et al., 2012; Wang et al., 2019 |
| 2-methylbutyl butanoate | 51115-64-1 | Wang et al., 2019 |
| 2-methylbutyl hexanoate | 2601-13-0 | Wang et al., 2019 |
| 2-methylpropyl 2-methylbutanoate | 2445-67-2 | Wang et al., 2019 |
| 2-methylpropyl acetate | 110-19-0 | Wang et al., 2019 |
| 2-methylpropyl butanoate | 539-90-2 | Wang et al., 2019 |
| 2-methylpropyl hexanoate | 105-79-3 | Wang et al., 2019 |
| butyl 2-methylbutanoate | 15706-73-7 | Wang et al., 2019 |
| butyl 2-methylpropanoate | 97-87-0 | Wang et al., 2019 |
| ethyl 2-methylbut-2-enoate | 5837-78-5 | Qin et al., 2012; Wang et al., 2019 |
| ethyl 2-methylbutanoate | 7452-79-1 | Qin et al., 2012; Wang et al., 2019 |
| ethyl 2-methylpropanoate | 97-62-1 | Qin et al., 2012; Wang et al., 2019 |
| hexyl 2-methylbutanoate | 10032-15-2 | Wang et al., 2019 |
| hexyl 2-methylprop-2-enoate | 142-09-6 | Wang et al., 2019 |
| hexyl 2-methylpropanoate | 2349-07-7 | Wang et al., 2019 |
| methyl 2-methylbutanoate | 868-57-5 | Qin et al., 2012; Wang et al., 2019 |
| propyl 2-methylbutanoate | 37064-20-3 | Wang et al., 2019 |
| Pequi |  |  |
| 2-methylbutyl hexanoate | 2601-13-0 | Belo et al., 2013 |
| 2-methylpropyl hexanoate | 105-79-3 | Maia et al., 2008; Belo et al., 2013; dos Santos da Silva et al., 2020 |
| 2-methylpropyl octadecanoate | 646-13-9 | Maia et al., 2008 |
| 3-methylbutyl 2-methylpropanoate | 2050-01-3 | Maia et al., 2008 |
| 3-methylbutyl 3-methylbutanoate | 659-70-1 | Belo et al., 2013 |
| 3-methylbutyl butanoate | 106-27-4 | dos Santos da Silva et al., 2020 |
| 3-methylbutyl hexanoate | 2198-61-0 | Maia et al., 2008; Belo et al., 2013 |
| 3-methylbutyl octanoate | 2035-99-6 | Belo et al., 2013 |
| ethyl 2-methylbutanoate | 7452-79-1 | Maia et al., 2008; Belo et al., 2013 |
| ethyl 2-methylpropanoate | 97-62-1 | Maia et al., 2008; dos Santos da Silva et al., 2020 |
| ethyl 3-methylbutanoate | 108-64-5 | Maia et al., 2008 |
| methyl 3-methylbutanoate | 556-24-1 | dos Santos da Silva et al., 2020 |
| propyl 3-methylbutanoate | 557-00-6 | Maia et al., 2008 |
| Pineapple |  |  |
| 2-methylpropyl acetate | 110-19-0 | Mohd Ali et al., 2020 |
| 3-methyl-1-butanol | 123-51-3 | Mohd Ali et al., 2020 |
| 3-methylbutyl acetate | 123-92-2 | Montero-Calderón et al., 2010 |
| ethyl 2-methylbutanoate | 7452-79-1 | Montero-Calderón et al., 2010; Zheng et al., 2012; Mohd Ali et al., 2020 |
| ethyl 2-methylpropanoate | 97-62-1 | Montero-Calderón et al., 2010 |
| methyl 2-hydroxy-2-methylbutanoate | 32793-34-3 | Zheng et al., 2012 |
| methyl 2-methylbutanoate | 868-57-5 | Montero-Calderón et al., 2010; Zheng et al., 2012; Mohd Ali et al., 2020 |
| methyl 2-methylpropanoate | 547-63-7 | Montero-Calderón et al., 2010; Mohd Ali et al., 2020 |
| methyl 3-methylbutanoate | 556-24-1 | Montero-Calderón et al., 2010 |
| Pitanga |  |  |
| 2-methylpropyl acetate | 110-19-0 | Oliveira et al., 2006 |
| 3-methylbutanal | 590-86-3 | Silva et al., 2019 |
| 3-methylbutyl acetate | 123-92-2 | Oliveira et al., 2006; Silva et al., 2019 |
| Pitaya |  |  |
| 2-methylpropyl acetate | 110-19-0 | Santos et al., 2020 |
| 3-methyl-1-butanol | 123-51-3 | Santos et al., 2020 |
| 3-methylbutyl acetate | 123-92-2 | Santos et al., 2020 |
| ethyl 3-methylbutanoate | 108-64-5 | Santos et al., 2020 |
| Pitomba |  |  |
| 3-methylbutyl acetate | 123-92-2 | de Souza et al., 2016 |
| Plum |  |  |
| 2-methyl-1-butanol | 137-32-6 | Pino and Quijano, 2012 |
| 2-methylbutanoic acid | 116-53-0 | Pino and Quijano, 2012 |
| 2-methylpropyl acetate | 110-19-0 | Pino and Quijano, 2012 |
| 3-methyl-1-butanol | 123-51-3 | Chai et al., 2012 |
| 3-methylbutanoic acid | 503-74-2 | Pino and Quijano, 2012 |
| 3-methylbutyl hexanoate | 2198-61-0 | Pino and Quijano, 2012 |
| butyl 2-methylbutanoate | 15706-73-7 | Pino and Quijano, 2012 |
| ethyl 2-methylbutanoate | 7452-79-1 | Pino and Quijano, 2012 |
| geranyl 3-methylbutanoate | 109-20-6 | Chai et al., 2012 |
| hexyl 2-methylbutanoate | 10032-15-2 | Chai et al., 2012; Pino and Quijano, 2012 |
| Pomegranate |  |  |
| 3-methyl-1-butanol | 123-51-3 | Güler and Gül, 2017 |
| 3-methylbutanal | 590-86-3 | Güler and Gül, 2017 |
| 3-methylbutanoic acid | 503-74-2 | Güler and Gül, 2017 |
| 3-methylbutyl acetate | 123-92-2 | Güler and Gül, 2017 |
| hexyl 2-methylpropanoate | 2349-07-7 | Güler and Gül, 2017 |
| Prickly pear fruit |  |  |
| methyl 2-methylbutanoate | 868-57-5 | Arena et al., 2001 |
| Pulasan |  |  |
| 3-methyl-1-butanol | 123-51-3 | Wong et al., 1996c |
| 3-methylbutanal | 590-86-3 | Wong et al., 1996c |
| Pumpkin |  |  |
| 2-methyl-1-butanol | 137-32-6 | Leffingwell et al., 2015 |
| 2-methyl-1-propanol | 78-83-1 | Leffingwell et al., 2015 |
| 2-methylbutanal | 96-17-3 | Leffingwell et al., 2015 |
| 2-methylpropanal | 78-84-2 | Leffingwell et al., 2015 |
| 3-methyl-1-butanol | 123-51-3 | Leffingwell et al., 2015 |
| 3-methylbutanal | 590-86-3 | Leffingwell et al., 2015 |
| Quince |  |  |
| 2-methyl-1-butanol | 137-32-6 | Tateo and Bononi, 2010 |
| 2-methylbutyl acetate | 624-41-9 | Tateo and Bononi, 2010 |
| 2-methylpropyl 2-methylbut-2-enoate | 7779-81-9 | Tateo and Bononi, 2010 |
| 2-methylpropyl acetate | 110-19-0 | Tateo and Bononi, 2010 |
| 2-methylpropyl hexanoate | 105-79-3 | Tateo and Bononi, 2010 |
| 2-methylpropyl octanoate | 5461-06-3 | Tateo and Bononi, 2010 |
| 3-methylbutyl acetate | 123-92-2 | Tateo and Bononi, 2010 |
| ethyl 2-methylbut-2-enoate | 5837-78-5 | Tateo and Bononi, 2010 |
| ethyl 2-methylbutanoate | 7452-79-1 | Tateo and Bononi, 2010 |
| hexyl 2-methylbutanoate | 10032-15-2 | Tateo and Bononi, 2010 |
| Rambai |  |  |
| methyl 2-hydroxy-3-methylbutanoate | 17417-00-4 | Wong et al., 1994 |
| methyl 2-methylbutanoate | 868-57-5 | Wong et al., 1994 |
| methyl 3-hydroxy-3-methylbutanoate | 6149-45-7 | Wong et al., 1994 |
| Rambutan |  |  |
| 2-methylbutanoic acid | 116-53-0 | Wong et al., 1996c; Ong et al., 1998 |
| 2-methylpropanoic acid | 79-31-2 | Ong et al., 1998 |
| 2-methylpropyl acetate | 110-19-0 | Ong et al., 1998 |
| 3-methyl-1-butanol | 123-51-3 | Wong et al., 1996c |
| 3-methylbutyl acetate | 123-92-2 | Wong et al., 1996c; Ong et al., 1998 |
| ethyl 2-methylbutanoate | 7452-79-1 | Ong et al., 1998 |
| ethyl 3-hydroxy-3-methylbutanoate | 18267-36-2 | Ong et al., 1998 |
| Raspberry |  |  |
| 2-methyl-1-butanol | 137-32-6 | Aprea et al., 2015 |
| 2-methyl-1-propanol | 78-83-1 | Aprea et al., 2015 |
| 2-methylbutanal | 96-17-3 | Aprea et al., 2015 |
| 2-methylbutane | 78-78-4 | Aprea et al., 2015 |
| 2-methylbutanoic acid | 116-53-0 | Aprea et al., 2015 |
| 2-methylbutyl acetate | 624-41-9 | Aprea et al., 2015 |
| 2-methylpropanal | 78-84-2 | Aprea et al., 2015 |
| 2-methylpropanoic acid | 79-31-2 | Aprea et al., 2015 |
| 3-methyl-1-butanol | 123-51-3 | Aprea et al., 2015 |
| 3-methylbutanal | 590-86-3 | Aprea et al., 2015 |
| 3-methylbutanoic acid | 503-74-2 | Aprea et al., 2015 |
| 3-methylbutyl acetate | 123-92-2 | Aprea et al., 2015 |
| ethyl 2-methylbutanoate | 7452-79-1 | Aprea et al., 2015 |
| ethyl 2-methylpropanoate | 97-62-1 | Aprea et al., 2015 |
| ethyl 3-methylbutanoate | 108-64-5 | Aprea et al., 2015 |
| Rata |  |  |
| 3-methyl-1-butanol | 123-51-3 | Pino et al., 2003b |
| Redcurrant |  |  |
| 2-(2-methylpropyl)-3-methoxypyrazine | 24683-00-9 | Jung, 2018 |
| 2-methyl-1-butanol | 137-32-6 | Jung, 2018 |
| 2-methyl-1-propanol | 78-83-1 | Jung, 2018 |
| 2-methylbutanoic acid | 116-53-0 | Jung, 2018 |
| 2-methylpropanoic acid | 79-31-2 | Jung, 2018 |
| 3-methyl-1-butanol | 123-51-3 | Jung, 2018 |
| Sapopdilla |  |  |
| 2-methyl-1-propanol | 78-83-1 | Pino et al., 2003a; Uekane et al., 2017 |
| 2-methylbutyl acetate | 624-41-9 | Uekane et al., 2017 |
| 2-methylpropanoic acid | 79-31-2 | Pino et al., 2003a |
| 2-methylpropyl acetate | 110-19-0 | Uekane et al., 2017 |
| 3-methyl-1-butanol | 123-51-3 | Pino et al., 2003a; Uekane et al., 2017 |
| 3-methylbutyl acetate | 123-92-2 | Uekane et al., 2017 |
| Sapota-do-Solimões |  |  |
| 2-methyl-1-propanol | 78-83-1 | Monteiro et al., 2018 |
| 2-methylbutanal | 96-17-3 | Monteiro et al., 2018 |
| 2-methylpropanal | 78-84-2 | Monteiro et al., 2018 |
| 3-methyl-1-butanol | 123-51-3 | Monteiro et al., 2018 |
| 3-methylbutanal | 590-86-3 | Monteiro et al., 2018 |
| 3-methylbutyl acetate | 123-92-2 | Monteiro et al., 2018 |
| ethyl 2-methylbut-2-enoate | 5837-78-5 | Monteiro et al., 2018 |
| ethyl 2-methylbutanoate | 7452-79-1 | Monteiro et al., 2018 |
| ethyl 2-methylpropanoate | 97-62-1 | Monteiro et al., 2018 |
| Shadberry |  |  |
| 3-methylbutanal | 590-86-3 | Parliament and Smith, 1999 |
| Siriguela |  |  |
| 2-methyl-1-butanol | 137-32-6 | Sosa-Moguel et al., 2018 |
| 2-methyl-1-propanol | 78-83-1 | Sosa-Moguel et al., 2018 |
| 2-methylbutanal | 96-17-3 | Sosa-Moguel et al., 2018 |
| 2-methylpropyl 2-methylbut-2-enoate | 7779-81-9 | Augusto et al., 2000 |
| 2-methylpropyl butanoate | 539-90-2 | Sosa-Moguel et al., 2018 |
| 3-methyl-1-butanol | 123-51-3 | Augusto et al., 2000 |
| 3-methylbutanal | 590-86-3 | Sosa-Moguel et al., 2018 |
| 3-methylbutyl acetate | 123-92-2 | Kozioł and Macía, 1998; Augusto et al., 2000 |
| 3-methylbutyl butanoate | 106-27-4 | Sosa-Moguel et al., 2018 |
| butyl 2-methylbut-2-enoate | 7785-66-2 | Ceva-Antunes et al., 2006 |
| butyl 3-methylbutanoate | 109-19-3 | Sosa-Moguel et al., 2018 |
| ethyl 2-methylbut-2-enoate | 5837-78-5 | Sosa-Moguel et al., 2018 |
| ethyl 2-methylbutanoate | 7452-79-1 | Augusto et al., 2000; Sosa-Moguel et al., 2018 |
| ethyl 2-methylpropanoate | 97-62-1 | Sosa-Moguel et al., 2018 |
| ethyl 3-methylbutanoate | 108-64-5 | Sosa-Moguel et al., 2018 |
| methyl 3-methylbutanoate | 556-24-1 | Sosa-Moguel et al., 2018 |
| propyl 2-methylbut-2-enoate | 61692-83-9 | Ceva-Antunes et al., 2006 |
| Snakefruit |  |  |
| 2-methylbut-2-enoic acid | 13201-46-2 | Wijaya et al., 2005 |
| 2-methylbutanoic acid | 116-53-0 | Wijaya et al., 2005 |
| methyl 2-methylbutanoate | 868-57-5 | Wijaya et al., 2005 |
| methyl 3-methylbut-2-enoate | 924-50-5 | Wijaya et al., 2005 |
| Soursop |  |  |
| 3-methylbutyl acetate | 123-92-2 | Márquez et al., 2011 |
| butyl 2-methylpropanoate | 97-87-0 | de Santana et al., 2017 |
| butyl 3-methylbutanoate | 109-19-3 | Leite Neta et al., 2019 |
| geranyl 3-methylbutanoate | 109-20-6 | Leite Neta et al., 2019 |
| methyl 2-hydroxy-3-methylbutanoate | 17417-00-4 | Cheong et al., 2010; Leite Neta et al., 2019 |
| Starfruit |  |  |
| 2-(2-methylpropyl)-3-methoxypyrazine | 24683-00-9 | Mahattanatawee‬ et al., 2005 |
| ethyl 2-methylbutanoate | 7452-79-1 | Mahattanatawee et al., 2005 |
| Strawberry |  |  |
| 2-methyl-3-hydroxypropanoic acid | 2068-83-9 | Oz et al., 2016 |
| 2-methylbutanoic acid | 116-53-0 | Ulrich et al., 2007 |
| 2-methylbutyl acetate | 624-41-9 | Ulrich et al., 2007 |
| 3-methylbutyl 2-methylpropanoate | 2050-01-3 | Misran et al., 2015 |
| 3-methylbutyl acetate | 123-92-2 | Ulrich et al., 2007; Misran et al., 2015 |
| 3-methylbutyl hexanoate | 2198-61-0 | Dong et al., 2013; Misran et al., 2015 |
| butyl 3-methylbutanoate | 109-19-3 | Misran et al., 2015 |
| ethyl 2-methylbutanoate | 7452-79-1 | Ulrich et al., 2007 |
| ethyl 3-methylbutanoate | 108-64-5 | Misran et al., 2015 |
| hexyl 3-methylbutanoate | 10032-13-0 | Misran et al., 2015; Oz et al., 2016 |
| methyl 2-methylbutanoate | 868-57-5 | Ulrich et al., 2007 |
| methyl 3-methylbutanoate | 556-24-1 | Misran et al., 2015 |
| octyl 2-methylbutanoate | 29811-50-5 | Oz et al., 2016 |
| Strawberry tree |  |  |
| 2-methyl-1-butanol | 137-32-6 | Oliveira et al., 2011 |
| 3-methyl-1-butanol | 123-51-3 | Oliveira et al., 2011 |
| Tamarillo |  |  |
| 2-methyl-1-butanol | 137-32-6 | Chen et al., 2020 |
| 2-methylbutanal | 96-17-3 | Chen et al., 2020 |
| 3-methyl-1-butanol | 123-51-3 | Chen et al., 2020 |
| Terebinth |  |  |
| 3-methylbutanoic acid | 503-74-2 | Amanpour et al., 2019 |
| Tomato |  |  |
| 2-(2-methylpropyl)-thiazole | 18640-74-9 | Baldwin et al., 2000; Tikunov et al., 2005; Tieman et al., 2012 |
| 2-methyl-1-butanol | 137-32-6 | Tikunov et al., 2005; Tieman et al., 2012 |
| 2-methylbutanal | 96-17-3 | Baldwin et al., 2000; Tikunov et al., 2005; Tieman et al., 2012 |
| 2-methylbutyl acetate | 624-41-9 | Tieman et al., 2012 |
| 2-methylpropyl acetate | 110-19-0 | Tieman et al., 2012 |
| 2-methylpropylnitrile | 78-82-0 | Baldwin et al., 2000 |
| 3-methyl-1-butanol | 123-51-3 | Baldwin et al., 2000; Tikunov et al., 2005; Tieman et al., 2012 |
| 3-methyl-1-nitrobutane | 627-67-8 | Baldwin et al., 2000; Tieman et al., 2012 |
| 3-methylbutanal | 590-86-3 | Baldwin et al., 2000; Tikunov et al., 2005; Tieman et al., 2012 |
| 3-methylbutanoic acid | 503-74-2 | Tikunov et al., 2005; Tieman et al., 2012 |
| 3-methylbutyl acetate | 123-92-2 | Tieman et al., 2012 |
| 3-methylbutylnitrile | 625-28-5 | Tieman et al., 2012 |
| 3-methylbutylnitrite | 110-46-3 | Tikunov et al., 2005 |
| Umbu |  |  |
| 2-methyl-1-propanol | 78-83-1 | de Sousa et al., 2010 |
| cinnamyl 3-methylbutanoate | 140-27-2 | de Sousa et al., 2010 |
| Uvaia |  |  |
| (Z)-3-hexenyl 2-methylpropanoate | 41519-23-7 | da Silva et al., 2019b |
| hexyl 2-methylpropanoate | 2349-07-7 | da Silva et al., 2019b |
| octyl 2-methylpropanoate | 109-15-9 | da Silva et al., 2019b |
| Uvilla |  |  |
| 2-methylpropanoic acid | 79-31-2 | Barrios Guio et al., 2010 |
| 2-phenoxyethyl 2-methylpropanoate | 103-60-6 | Barrios Guio et al., 2010 |
| 3-methyl-1-butanol | 123-51-3 | Barrios Guio et al., 2010 |
| 3-methylbutanoic acid | 503-74-2 | Barrios Guio et al., 2010 |
| Watermelon |  |  |
| 3-methylbutyl butanoate | 106-27-4 | Fredes et al., 2016 |
| ethyl 2-methylbutanoate | 7452-79-1 | Fredes et al., 2016 |
